# VEGF-A regulated by progesterone governs uterine angiogenesis and vascular remodelling during pregnancy

**DOI:** 10.1002/emmm.201302618

**Published:** 2013-08-02

**Authors:** Minah Kim, Hyeung Ju Park, Jae Won Seol, Jeon Yeob Jang, Young-Suk Cho, Kyu Rae Kim, Youngsok Choi, John P Lydon, Francesco J DeMayo, Masabumi Shibuya, Napoleone Ferrara, Hoon-Ki Sung, Andras Nagy, Kari Alitalo, Gou Young Koh

**Affiliations:** 1 Laboratory of Vascular Biology and Stem Cells and Graduate School of Medical Science and Engineering, Korea Advanced Institute of Science and Technology (KAIST)Daejeon, Korea; 2 College of Veterinary Medicine, Chonbuk National UniversityJeonju, Korea; 3 Department of Pathology, University of Ulsan College of Medicine, Asan Medical CenterSeoul, Korea; 4 Department of Biomedical Science, CHA UniversitySeoul, Korea; 5 Department of Molecular and Cellular Biology, Baylor College of MedicineHouston, TX, USA; 6 Institute of Physiology and Medicine, Jobu UniversityGunma, Japan; 7 Moores Cancer Center, University of California San DiegoLa Jolla, CA, USA; 8 Samuel Lunenfeld Research Institute, Mount Sinai HospitalToronto, Ontario, Canada; 9 Molecular/Cancer Biology Laboratory, Institute for Molecular Medicine, University of HelsinkiHelsinki, Finland

**Keywords:** decidual angiogenesis, pregnant uterus, vascular sinus folding, vascular regression, VEGF-A

## Abstract

The features and regulation of uterine angiogenesis and vascular remodelling during pregnancy are poorly defined. Here we show that dynamic and variable decidual angiogenesis (sprouting, intussusception and networking), and active vigorous vascular remodelling such as enlargement and elongation of ‘vascular sinus folding’ (VSF) and mural cell drop-out occur distinctly in a spatiotemporal manner in the rapidly growing mouse uterus during early pregnancy — just after implantation but before placentation. Decidual angiogenesis is mainly regulated through VEGF-A secreted from the progesterone receptor (PR)-expressing decidual stromal cells which are largely distributed in the anti-mesometrial region (AMR). In comparison, P_4_-PR-regulated VEGF-A-VEGFR2 signalling, ligand-independent VEGFR3 signalling and uterine natural killer (uNK) cells positively and coordinately regulate enlargement and elongation of VSF. During the postpartum period, Tie2 signalling could be involved in vascular maturation at the endometrium in a ligand-independent manner, with marked reduction of VEGF-A, VEGFR2 and PR expressions. Overall, we show that two key vascular growth factor receptors — VEGFR2 and Tie2 — strikingly but differentially regulate decidual angiogenesis and vascular remodelling in rapidly growing and regressing uteri in an organotypic manner.

## INTRODUCTION

Each organ has distinct and unique neovascularization and vascular remodelling processes for its growth, maintenance and regeneration. Understanding the regulation of organotypic neovascularization and vascular remodelling is essential and provides insight into how to control these processes. Neovascularization (including vasculogenesis and angiogenesis) and vascular remodelling (including branching, enlargement and network formation) are known to be majorly regulated by two key growth factor families and their cognate receptors: vascular endothelial growth factor (VEGF) and its receptor (VEGFR) and angiopoietin (Ang) and the Tie2 receptor (Augustin et al, 2009; Carmeliet, 2003; Chung & Ferrara, 2011; Lohela et al, 2009). However, we are only beginning to understand the organ-specific roles of these molecules.

The uterus is an exceptional organ, showing enormous cyclic neovascularization and vessel regression during the uterine cycle period, while neovascularization is mostly at rest during premenstrual and postmenopausal stages of life. In early pregnancy, the rapid growth of the uterus during embryo implantation and the early post-implantation period is accompanied by profound neovascularization and vascular remodelling to deliver sufficient oxygen and nutrients to the uterus as well as to the embryo until the placenta becomes structurally and functionally competent (Cha et al, 2012; Dey et al, 2004; Wang & Dey, 2006). Implantation of an embryo induces rapid proliferation and differentiation of uterine stromal cells, forming a new structure, the decidua (Cha et al, 2012; Dey et al, 2004; Ramathal et al, 2010; Wang & Dey, 2006). One salient feature of decidua formation is ‘decidual angiogenesis’, a marked increase in uterine neovascularization that is promoted during early pregnancy by factors secreted from decidual stromal cells (DSCs) and the adjacent immune cells, such as uterine natural killer (uNK) cells and dendritic cells (uDCs) (Blois et al, 2011; Demir et al, 2010; Plaisier, 2011; Ramathal et al, 2010; Rowe et al, 2003; Smith, 2001; Torry et al, 2007; Zhang et al, 2011). Decidual angiogenesis forms a new vascular network that serves as the first exchange apparatus between maternal circulation and the developing embryo, and thus is a crucial and fundamental process for embryonic survival and successful pregnancy. The steroid hormones progesterone (P4, pregn-4-ene-3,20-dione) and estrogen (E2, 17β-estradiol) are secreted from ovaries and, through binding of the progesterone receptor (PR) and estrogen receptor (ER), play orchestral roles in regulating decidua formation during early pregnancy (Carson et al, 2000; Conneely et al, 2003; Wetendorf & DeMayo, 2012). Two decades ago, Keshet's research group first suggested that high VEGF-A expression in DSCs could be regulated by steroid hormones (Shweiki et al, 1993). Since then, the roles of P4, E2 and their receptors in decidual angiogenesis have been studied, but they appear to vary depending on species, timing and experimental design (Das et al, 2009; Girling et al, 2007; Heryanto & Rogers, 2002; Walter et al, 2005), and thus have not yet been firmed established. Moreover, it is unknown how P4 and E2 affect the VEGF-A-VEGFR2 system in a spatiotemporal manner to promote decidual angiogenesis during early pregnancy.

It is known that the expressions of VEGF-VEGFR and Ang-Tie2 in the uterus dynamically change during the estrus cycle period, embryo implantation and the early post-implantation period (Chakraborty et al, 1995; Demir et al, 2010; Plaisier, 2011; Rowe et al, 2003; Smith, 2001; Torry et al, 2007). Functional studies indicate that VEGF-A — a major angiogenic growth factor and a ligand to VEGFR2 — is involved in regulating uterine angiogenesis and implantation in rodents and nonhuman primates (Heryanto & Rogers, 2002; Klauber et al, 1997; Sengupta et al, 2007; Walter et al, 2005). Moreover, a recent study using specific blockades reported that VEGFR2 signalling (but not VEGFR1 signalling) is critical in decidual angiogenesis, vascular remodelling and pregnancy progression, while VEGFR3 signalling may moderately contribute to decidual angiogenesis but has no effect on early pregnancy progression (Douglas et al, 2009). It remains poorly understood where and how VEGF-A is expressed and regulated, and how VEGF-A–VEGFR2 signalling drives decidual angiogenesis and vascular remodelling in a spatiotemporal manner during early pregnancy. Moreover, little is known regarding to what extent the driving force for decidual angiogenesis and vascular remodelling during early pregnancy can be attributed to VEGF-C/D-VEGFR3 signalling. Furthermore, during the postpartum period, the uterus undergoes rapid shrinkage with strong contractile activity and endometrium normalization, but little is known about the exact characteristics and regulations of vascular remodelling and regression in the endometrium.

In the present study, the spatiotemporal changes of decidual angiogenesis and vascular remodelling in the mouse uterus during the post-implantation period (from 4.5 to 8.5 days post coitum (dpc) before active placental development) and during the postpartum period were determined. We shed a light on the feature and regulation of VSF, arterio-venous vascular shunt or reservoir, which is markedly enlarged and elongated during this period. We found that the VEGF-A/VEGFR2 system plays a critical role in decidual angiogenesis and VSF remodelling, and thus we tried to clarify how VEGF-A expression is regulated in DSCs. We found that the P4-PR axis is a main regulator of VEGF-A expression in DSCs, while VEGFR3 and uNK cells are also involved in VSF remodelling. Moreover, when we determined how blood vessels are regressed and undergoing vascular maturation in the endometrium during the postpartum period, we unexpectedly found indications of the ligand-independent involvement of Tie2 signalling with marked reductions of expressions of VEGF-A, VEGFR2 and PR, and restoration of mural cell coverage on the blood vessels.

## RESULTS

### Dynamic but distinct uterine angiogenesis and vascular remodelling at different regions during early pregnancy

**Figure 1:**
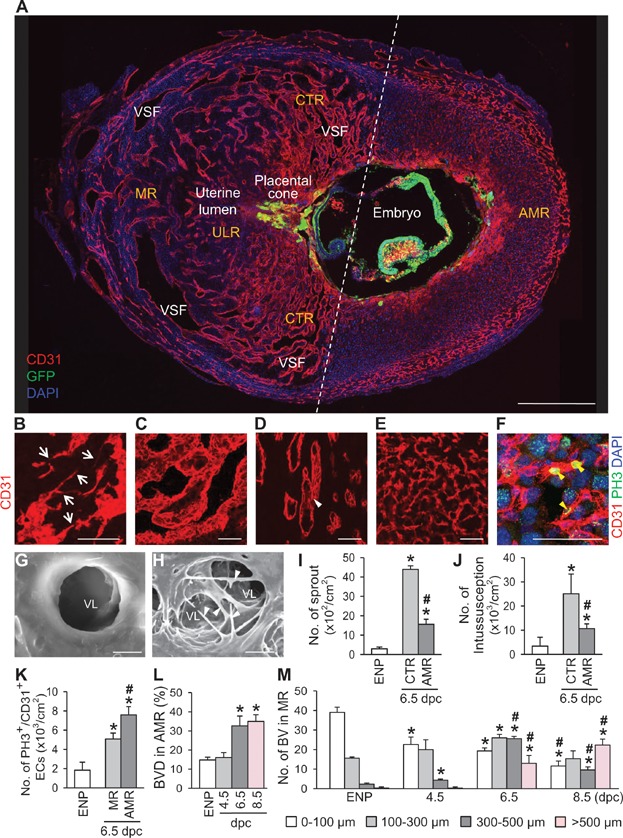
Distinct regional differences in uterine vascular features during early pregnancy **A.** Image showing CD31^+^ blood vessels (BVs) and GFP^+^ embryo in the uterus at 8.5 dpc. Dotted-line divides the mid-sectioned uterus into the mesometrial region (MR) and anti-mesometrial region (AMR). The MR is subdivided into the central region (CTR) and uterine lumen region (ULR). VSF, vascular sinus folding. Scale bar, 500 µm.**B–F.** Images showing CD31^+^ uterine BVs. Sprouting (B, arrows), variable-sized VSF (C) and intussusceptive pillar (D, white arrowheads) are frequently present in the MR, whereas fine vascular network (E) and PH3^+^ proliferating ECs (F, yellow arrowheads) are present in the AMR at 6.5 dpc. Scale bars, 50 µm.**G, H.** Scanning electron micrographs showing vascular lumens (VL) without and with abundant intussusceptive pillars (arrowheads) in the MR at 6.5 dpc. Scale bars, 5 µm.**I, J.** Comparisons of numbers of vascular sprouts and intussusceptions in the uterine endometrium of the estrus stage of non-pregnancy (ENP), and the CTR and AMR at 6.5 dpc. Each group, *n* = 5–6. *p < 0.002 versus ENP.; ^#^*p* < 0.001 versus MR by unpaired *t* test.**K.** Comparisons of number of PH3^+^/CD31^+^ proliferative ECs in the uterine endometrium of the ENP, and the MR and AMR at 6.5 dpc in a given area (cm^2^). Each group, *n* = 5–6 *p < 0.0003 versus ENP; ^#^*p* = 0.0014 versus MR by unpaired *t* test.**L, M.** Comparisons of CD31^+^ BV densities (BVD, %) in the AMR and numbers of different sized VSFs in the MR at ENP, and 4.5, 6.5, and 8.5 dpc. Each group, *n* = 5–6. *p < 0.02 versus ENP.; ^#^*p* < 0.02 versus 4.5 dpc by one-way ANOVA.

Taking advantage of profound morphological changes in the mouse uterus during early pregnancy (Supporting Information Fig S1A and B), we analysed structural changes in blood vessels and the molecular regulation of angiogenesis and vascular remodelling in the uterus from 4.5 to 8.5 dpc, which is just after implantation (4.0–4.5 dpc) but prior to initial placenta establishment (9.5–10.5 dpc). To distinguish maternal uterine blood vessels from placental blood vessels, a set of B6 females were mated with syngeneic GFP^+^ males, and the pregnant uteri were obtained at 4.5, 6.5 and 8.5 dpc. The GFP^+^ embryos were easily distinguishable from the maternal uteri at each time point (Fig 1A and Supporting Information Fig S2A). The embryo-derived placental cone was clearly demarcated on 8.5 dpc, when the umbilical and vitelline vessels emerged (Fig 1A). Taking advantage of the clear demarcation, only uterine blood vessels were convincingly highlighted by immunovisualization of CD105 (endoglin) or CD31 in mid-sectioned uteri (Fig 1A). Variable-sized VSFs (Chandana et al, 2010) were distributed symmetrically in the central region (CTR) and were enlarged and elongated over time, whereas fine mesh-like blood vessels were arrayed in the anti-mesometrial region (AMR) (Fig 1A and Supporting Information Fig S1 and 2). Closer observations of the CTR revealed that the sprouting process was most active at 4.5 dpc, while enlargement and elongation of VSF (EEVSF) and intussusception of blood vessels (Burri et al, 2004) were dominant from 6.5–8.5 dpc (Fig 1B–E, G and H). At 6.5 dpc, number of vascular sprouts (>20 μm in length) in the CTR was increased by 14.9- and 2.8-fold compared to those in the estrus stage of non-pregnancy (ENP) and the AMR (Fig 1I). Moreover, number of intussusceptive blood vessels in the CTR was increased by 7.4- and 2.4-fold compared to those of the ENP and the AMR (Fig 1J). At 6.5 dpc, proliferating phosphohistone H3 (PH3)^+^ endothelial cells (ECs) in the AMR were elevated by 4.2- and 1.5-fold compared to those of the ENP and the MR (Fig 1F and K). Time-series analyses indicated that, compared to at 4.5 dpc, the number of large-sized (diameter, >300 μm) VSF in the CTR was markedly increased at 6.5 and 8.5 dpc, and blood vessel densities in the AMR were increased by 2.0- and 2.2-fold at 6.5 and 8.5 dpc, respectively (Fig 1L, M and Supporting Information Fig S1B). Moreover, the majority of blood vessels in the uteri were covered with α-smooth-muscle actin (α-SMA)-positive mural cells at 4.5 dpc; however, at 6.5 and 8.5 dpc, this coverage had largely disappeared in most regions, except the surrounding of spiral arteries (Supporting Information Fig S2B). Thus, profound variable decidual angiogenesis; sprouting, intussusception and networking angiogenesis with high EC proliferation, and dynamic vascular remodelling; EEVSF and mural cell drop-out occurred distinctly at different regions in the growing uterus during the post-implantation period before placenta establishment.

### The VEGF-A-VEGFR2 system plays a major role in decidual angiogenesis and EEVSF during early pregnancy

**Figure 2:**
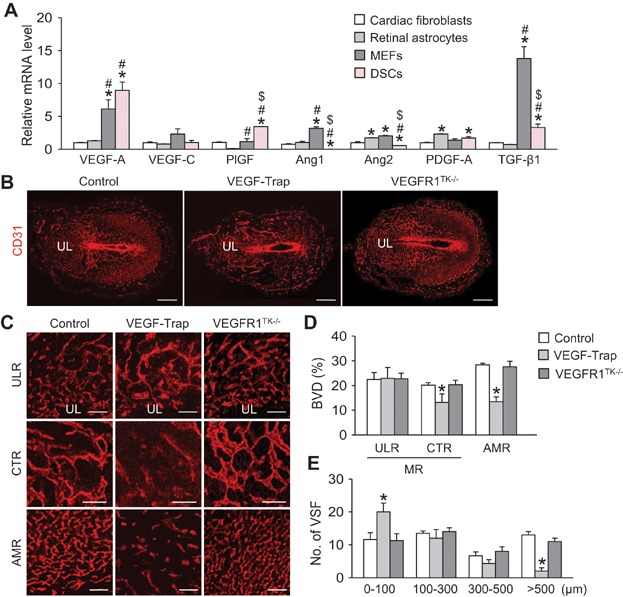
VEGF-A-VEGFR2 system is a key regulator of decidual angiogenesis and EEVSF during early pregnancy **A.** Comparisons of mRNA levels of genes regulating angiogenesis in primary cultured DSCs, cardiac fibroblasts, retinal astrocytes and mouse embryonic fibroblasts (MEFs). Data are presented as relative folds to the levels of cardiac fibroblasts after normalization with GAPDH. Each group, *n* = 4. *p < 0.05 versus cardiac fibroblasts; ^#^*p* < 0.04 versus retinal astrocytes; ^$^*p* < 0.03 versus MEFs by one-way ANOVA.**B.** Representative images showing CD31^+^ BVs in the uteri at 6.5 dpc of control, VEGF-Trap treated and VEGFR1^TK−/−^. Scale bars, 500 µm. UL, uterine lumen.**C.** Magnified images showing endometrial CD31^+^ BVs in the ULR, CTR of the MR and AMR. Scale bars, 100 µm.**D, E.** Comparisons of CD31^+^ BVD (%) in the ULR, CTR and AMR and numbers of different sized VSFs in the CTR at 6.5 dpc. Each group, *n* = 3–4. *p < 0.04 versus Control by one-way ANOVA.

These initial observations led us to investigate how dynamic vascular remodelling is finely regulated by key angiogenic growth factors during early pregnancy. DSCs are a main resource for a battery of angiogenic growth factors (Demir et al, 2010; Dey et al, 2004; Plaisier, 2011; Rowe et al, 2003; Torry et al, 2007); therefore we compared relative expression levels of the growth factors in primary cultured DSCs to those of other primary cultured stromal cells, such as cardiac fibroblasts, retinal astrocytes, and mouse embryonic fibroblasts (MEFs). Quantitative real-time PCR analyses revealed that, in DSCs compared to in other stromal cells, VEGF-A mRNA expression was higher, and mRNA expressions of placental growth factor (PlGF), angiopoietin-1 (Ang1), angiopoietin-2 (Ang2), VEGF-C, PDGF-A and TGF-β1 were slightly higher, similar, or lower (Fig 2A). Based on these findings, we examined role of VEGF-A in decidual angiogenesis and vascular remodelling in the pregnant uteri by treatment of VEGF-Trap (simultaneous blockade of VEGF-A and PlGF (Holash et al, 2002) at 4.5 dpc. This treatment resulted in profoundly pruned vascular structures and reduced blood vessel densities in both the CTR (35%) and AMR (53%) at 6.5 dpc compared to those of control treated mice (Fig 2B–D). Furthermore, embryo resorption was observed in close to half of the VEGF-Trap-treated mice (Supporting Information Table S1). We also noted that VEFG-Trap treatment markedly decreased the number of large-sized (>500 μm) VSF in the CTR (85%) (Fig 2C and E). Following VEGF-Trap treatment at 6.5 dpc, we observed similar reductions in blood vessel densities and numbers of large-sized VSF at 8.5 dpc, but without significant embryo resorption (Supporting Information Fig 3A–C and Supporting Information Table S1). We next used VEGFR1-tyrosine kinase-deficient (VEGFR1^TK−/−^) mice to investigate the role of PlGF, which only binds to VEGFR1 (Hiratsuka et al, 1998). In contrast to the findings with VEGF-Trap, blood vessel densities and vascular structures did not differ between wild-type and VEGFR1^TK−/−^ mice at 6.5 dpc (Fig 2B–D). Moreover, expressions of Ang1, Ang2 and Tie2 were detectable but relatively low in the DSCs and vasculatures of uteri at 6.5 dpc (data not shown). These data indicated that decidual angiogenesis during early pregnancy was mainly regulated through VEGF-A/VEGFR2 signalling, not through PlGF/VEGFR1 or Ang-Tie2 signalling. Moreover, VEGF-A-VEGFR2 signalling is positively involved in EEVSF.

### VEGF-A expression of DSCs is regulated in a spatiotemporal manner, but is independent of hypoxia

**Figure 3:**
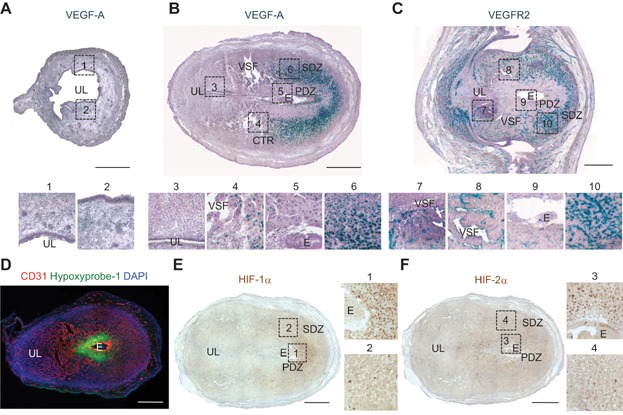
VEGF-A is highly expressed in the DSCs of pregnant uterus, but is not spatially matched with hypoxia and HIF-1α and HIF-2α expressions **A–C.** Images showing expressions of VEGF-A and VEGFR2 in the uteri of VEGF^+/LacZ^ and VEGFR2^+/LacZ^ mice at ENP (A) and 6.5 dpc (B and C). Each numbered region (square-dotted line) is magnified and arrayed in below. The tissues were counterstained with hematoxylin (violet) following X-gal staining. UL, uterine lumen; E, embryo; PDZ, primary decidual zone; SDZ, secondary decidual zone; CTR, the central region; VSF, vascular sinus folding. Scale bars, 500 µm.**D.** Image showing Hypoxyprobe-1^+^ hypoxic region and CD31^+^ BVs in uterus at 6.5 dpc. Scale bar, 500 µm.**E, F** Expressions of HIF-1α and HIF-2α in the uteri at 6.5 dpc. Each numbered region (square-dotted line) is magnified and arrayed in right side. Scale bars, 500 µm.

To explore the regulatory role of VEGF-A/VEGFR2 signalling in decidual angiogenesis and EEVSF, we first examined VEGF-A expression in the pregnant uterus of VEGF-A-LacZ reporter (VEGF-A^+/LacZ^) mice (Miquerol et al, 2000). The spatial pattern of VEGF-A expression in the uterus was quite striking at 6.5 dpc, in that it was highly expressed in DSCs at the secondary decidual zone of the AMR and in surrounding regions of the enlarged VSF, but its expression was largely absent or barely detectable in most regions of the MR and ENP (Fig 3A and B). Immunohistochemical analysis revealed that secreted VEGF-A appeared to be bound to vascular matrix or VEGFR2 at the secondary decidual zone of the AMR (Supporting Information Fig 3D). In comparison, during early pregnancy, VEGFR2 was expressed in all vasculatures of most regions, including endometrium and myometrium (Fig 3C and Supporting Information Fig 3E). These data implied that VEGF-A expression was finely regulated in a spatiotemporal manner, and that VEGFR2 signalling in the pregnant uterus could be activated through both ligand-dependent and ligand-independent manners. Because hypoxia is the strongest stimulus for VEGF-A expression (Ferrara, 2004), we determined spatial correlations among VEGF-A expression, hypoxia extent, and expressions of hypoxia-inducible factor-1α (HIF-1α) and HIF-2α in the uteri at 6.5 dpc. Hypoxia extent, HIF-1α expression and HIF-2α expression were not correlated with VEGF-A expression; however, there were spatial correlations between hypoxia and expressions of HIF-1α and HIF-2α at the primary decidual zone (Fig 3D–F). Thus, regulation of VEGF-A expression in the DSCs of the pregnant uterus seemed to be independent of hypoxia.

### P4-PR axis positively regulates VEGF-A expression of DSCs for decidual angiogenesis and EEVSF

**Figure 4:**
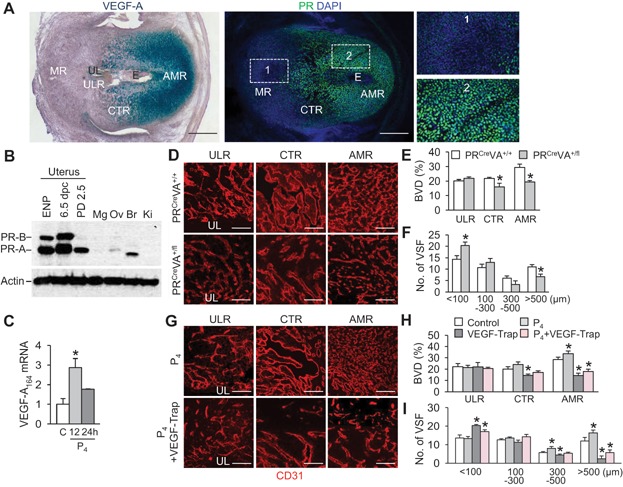
P4-PR axis positively regulates VEGF-A expression in DSCs for decidual angiogenesis and EEVSF **A.** Comparison of VEGF-A expression and PR expression in the uteri at 6.5 dpc. Each numbered region (square-dotted line) is magnified in right side. UL, uterine lumen; E, embryo. Scale bars, 500 µm.**B.** Comparisons of PR-A and PR-B protein levels in the uteri of EPN, 6.5 dpc and PD 2.5, and in mammary glands (Mg), ovary (Ov), brain (Br) and kidney (Ki) in 8-week-old non-pregnant female mouse. Loading of similar amount of protein for each sample is verified by a similar intensity of β-actin signal.**C.** Increase of VEGF-A mRNA expression in the primary cultured DSCs by P4 (10 µM) stimulation for 12 and 24 h. Data are presented as relative fold to the control (C) after normalization with GAPDH. Each group, *n* = 3. *p = 0.0232 versus C by unpaired *t* test.**D.** Images showing CD31^+^ BVs in the ULR, CTR and AMR of control PR-Cre-VEGF-A^+/+^ mice (PR^Cre^VA^+/+^) and PR-Cre-VEGF-A^+/fl^ (PR^Cre^VA^+/fl^) mice at 6.5 dpc. Scale bars, 100 µm.**E, F.** Comparisons of CD31^+^ BVD (%) in the ULR, CTR and AMR, and numbers of different sized VSFs in the CTR of control PR^Cre^VA^+/+^ and PR^Cre^VA^+/fl^ mice at 6.5 dpc. Each group, *n* = 3. *p < 0.03 versus PR^Cre^VA^+/+^ by one-way ANOVA.**G.** Images comparing CD31^+^ BVs in the ULR, CTR and AMR at 6.5 dpc treated P4 and P4 plus VEGF-Trap. Scale bars, 100 µm.**H, I.** Comparisons of CD31^+^ BVD (%) in the ULR, CTR and AMR, and numbers of different sized VSFs in the CTR at 6.5 treated with control, P4, VEGF-Trap and P4 plus VEGF-Trap. Each group, *n* = 3. *p < 0.05 versus control by one-way ANOVA.

We next examined the spatial correlation between VEGF-A expression and PR expression since ovary-secreted P4 and the DSC-localized PR axis are known to play critical roles in decidua formation and angiogenesis during early pregnancy (Conneely et al, 2003; Dey et al, 2004; Lydon et al, 1995; Wetendorf & DeMayo, 2012). Indeed, PR was mainly expressed in DSCs of the CTR and AMR, and expressions of VEGF-A and PR spatiotemporally coincided (Fig 4A). We also noted that two forms of PR — PR-A and PR-B (Conneely et al, 2003) — were highly detected in the normal uterus compared to in other organs, including mammary gland, ovary, brain and kidney; their levels were up-regulated at 6.5 dpc, but largely reduced at postpartum day 2.5 (PD 2.5) (Fig 4B). In primary cultured DSCs, mRNA expression of the major form of VEGF-A (VEGF-A_164_) was increased by addition of 10 µM P4 (Fig 4C). Compared to control PR-Cre-VEGF-A^+/+^ mice at 6.5 dpc, in PR-Cre-VEGF-A^+/flox^ mice (with one copy of the VEGF-A gene depleted in the DSCs), blood vessels were moderately pruned, blood vessel densities were reduced in both the CTR (26%) and AMR (32%) regions but not in the uterine lumen region (ULR), and the numbers of 300–500 μm (44%) and >500 μm (45%) VSF were reduced in the CTR (Fig 4D–F). Conversely, supplemental P4 (20 mg/kg) administered at 4.5 and 5.5 dpc increased blood vessel densities in both the CTR (21%) and AMR (18%) but not in the ULR, and increased numbers of 300–500 μm (41%) and >500 μm (36%) VSF in the CTR compared to in control mice at 6.5 dpc (Fig 4G–I). Blockade of VEGF-A by administration of VEGF-Trap (twice, at 4.5 and 5.5 dpc) completely abrogated the P4-induced increases in blood vessel densities and EEVSF (Fig 4G–I), indicating that the P4-induced decidual angiogenesis and EEVSF in the pregnant uterus were mainly mediated through up-regulation of VEGF-A expression in DSCs.

**Figure 5:**
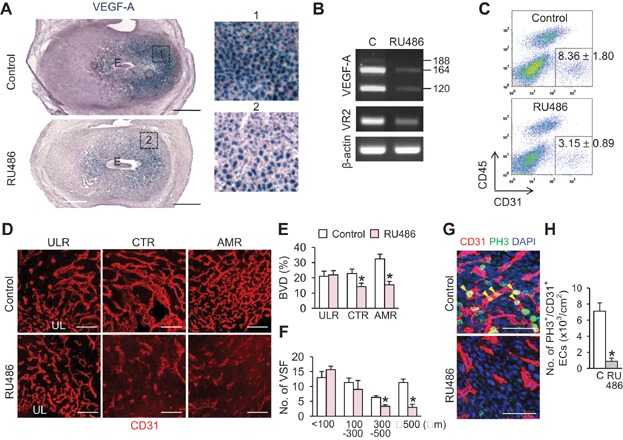
Pharmacologic blockade of PR with RU486 negatively regulates VEGF-A expression in DSCs for decidual angiogenesis and EEVSF The pregnant mice were treated with control buffer (Control, C) and RU486 (8 mg/kg) at 5.5 dpc, and the uteri were harvested at 6.5 dpc and analysed. **A.** Comparison of VEGF-A expression in the uteri of VEGF^+/LacZ^ mice. Each numbered region (square-dotted line) is magnified in right side. Scale bars, 500 µm.**B.** Semi-quantitative RT-PCR showing mRNA expressions of VEGF-A and VEGFR2 (VR2). Each line indicates gene transcript of VEGF-A_120_, VEGF-A_164_ and VEGF-A_188_.**C.** FACS analysis showing CD31^+^/CD45^−^ EC populations from the endometrium. Each group, *n* = 4.**D.** Images comparing CD31^+^ BVs in the ULR, CTR and AMR. Scale bars, 100 µm.**E, F** Comparisons of CD31^+^ BVD (%) in the ULR, CTR and AMR, and numbers of different sized VSFs in the CTR. Each group, *n* = 4. *p < 0.01 versus Control by one-way ANOVA.**G.** Images showing PH3^+^ proliferative ECs (yellow arrowheads) in the AMR. Scale bars, 100 µm.**H.** Comparison of number of PH3^+^/CD31^+^ proliferative ECs in a given area (cm^2^) of the endometrium. Each group, *n* = 4 *p = 0.001 versus C by unpaired *t* test.

To verify this functional role of the P4-PR-VEGF-A axis in decidual angiogenesis and EEVSF, we performed a pharmacological blockade of PR in pregnant mice by a single administration of RU486 (8.0 mg/kg) at 5.5 dpc, determined based on our preliminary trials for selection of timing and dosage of RU486. As expected, administration of RU486 at 5.5 dpc reduced expressions of VEGF-A and VEGFR2, number of CD31^+^/CD45^−^ EC (62%), blood vessel densities in both the CTR (38%) and AMR (53%) but not in the ULR, numbers of 300–500 μm (48%) and >500 μm (74%) VSF in the CTR, and EC proliferation rate (88%) in the AMR in the treated uteri compared to in the controls at 6.5 dpc (Fig 5); RU486 treatment also led to embryo resorption in almost 30% of mice (Supporting Information Table S1). To exclude possible contribution of ovarian hormones other than progesterone in decidual angiogenesis and EEVSF, we conducted a bilateral ovariectomy with supplementation of P4 according to previous reports (Douglas et al, 2009; Walter et al, 2005). At 6.5 dpc, we found no significant differences of vascular densities and structures in the pregnant uteri in the ovariectomized P4-supplemented mice compared to in sham-operated control mice (Supporting Information Fig S4A–C). To determine the effect of E2 on decidual angiogenesis and EEVSF, we administrated the ER inhibitor ICI 182780 (1 mg/kg) at 5.5 dpc. At 6.5 dpc, we found no significant differences in the vascular densities and structures in the pregnant uteri of ICI 182780-treated mice compared to control-treated mice (Supporting Information Fig S4D–F). Thus, the ovary-secreted P4/PR-expressing DSC axis positively and spatially governed VEGF-A expression in DSCs for decidual angiogenesis at the AMR and EEVSF, particularly at the CTR, during early pregnancy after implantation.

### Possible involvement of VEGFR3 and uNK cells, but not uDCs, in EEVSF during post-implantation period

**Figure 6:**
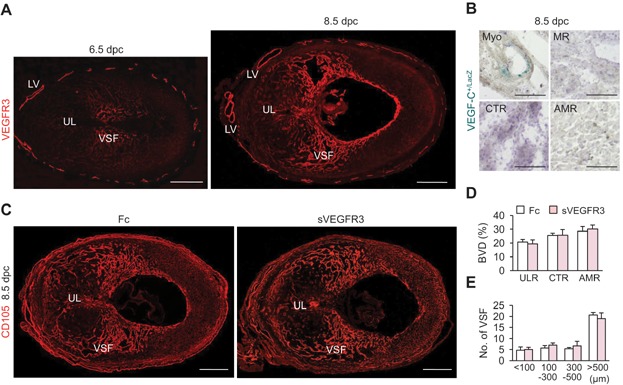
VEGFR3 is selectively and profoundly expressed in the EC of VSFs at the CTR and could be involved in EEVSF in a ligand independent manner during early pregnancy **A.** Representative images showing VEGFR3^+^ VSFs in the endometrium and VEGFR3^+^ lymphatic vessels (LV) in the myometrium of uteri at 6.5 and 8.5 dpc. Scale bars, 500 µm. UL, uterine lumen.**B.** Images showing VEGF-C expression in the myometrium (Myo), and the MR, CTR and AMR of endometrium in uterus of VEGF-C^+/LacZ^ mouse at 8.5 dpc. Scale bars, 100 µm.**C.** Images showing CD105^+^ BVs in the uteri at 8.5 dpc treated with Fc and soluble VEGFR3 (sVEGFR3, 25 mg/kg). Scale bars, 500 µm.**D, E.** Comparisons of CD105^+^ BVD (%) in the ULR, CTR and AMR, and numbers of different sized of VSFs in the CTR at 8.5 dpc treated with Fc and sVEGFR3. Each group, *n* = 4.

**Figure 7:**
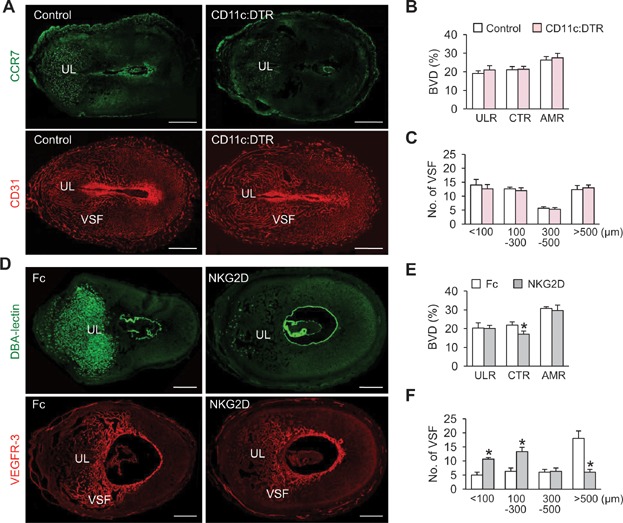
uNK cells, but not uDC, could be involved in EEVSF during post-implantation period **A.** Images showing CCR7^+^ uDCs and CD31^+^ BVs in the uteri of DTR (Control) and CD11c:DTR mice at 6.5 dpc after DTx treatment at 5.5 dpc. Scale bars, 500 µm. UL, uterine lumen.**B, C.** Comparisons of CD31^+^ BVD (%) in the ULR, CTR and AMR, and numbers of different sized VSFs in the CTR at 6.5 dpc in Control and CD11c:DTR mice. Each group, *n* = 4.**D.** Images showing DBA-lectin^+^ uNK cells and VEGFR3^+^ VSFs in the uteri at 8.5 treated with Fc and anti-NKG2D antibody. Scale bars, 500 µm.**E, F.** Comparisons of CD31^+^ BVD (%) in the ULR, CTR and AMR, and numbers of different sized VSFs in the CTR at 8.5 dpc treated with Fc and anti-mouse NKG2D antibody. Each group, *n* = 4. *p < 0.03 versus Fc by one-way ANOVA.

VEGFR3, the cognate receptor of VEGF-C/D, together with VEGFR2, was strongly expressed in the ECs of the VSF and the Prox-1+ lymphatic vessels of early pregnant uteri (Fig 6A and Supporting Information Fig S5), although VEGF-C expression was barely detectable in the adjacent tissues (Fig 6B); therefore, we next determined the role of VEGFR3 in vascular remodelling by inducing VEGF-C/D blockade through twice administrations of sVEGFR3-Fc (25 mg/kg) at 6.5 and 7.5 dpc. Vascular densities and structures in three different regions at 8.5 dpc were not distinguishable between sVEGFR3-Fc- and Fc-administered mice (Fig 6C–E). Thus, VEGFR3 may be involved in EEVSF in a ligand-independent manner (Benedito et al, 2012) or via heterodimerization with VEGFR2 (Nilsson et al, 2010; Wang et al, 2010). To determine the role of uDCs on vascular remodelling, CD11c:DTR transgenic mice (Jung et al, 2002) were treated with diphtheria toxin (DTx) at 5.5 or 6.5 dpc (Fig 7A). Vascular densities and structures in three different regions at 6.5 or 8.5 dpc were not distinguishable between uDC-depleted mice and uDC-intact mice (Fig 7A–C). However, we detected impaired vascular remodelling and embryonic resorption at 6.5 dpc when DTx was administrated at 4.5 dpc (Supporting Information Table S1), showing phenotypes consistent with those previously reported (Plaks et al, 2008). These results indicated that uDCs had a crucial role in establishing embryo implantation, but not in the uterine vascular remodelling occurring after successful embryo implantation. To determine the role of uNK cells-secreted growth factors on vascular remodelling, uNK cells were depleted by administration of anti-NKG2D antibody (7.5 mg/kg) at 6.5 and 7.5 dpc (Fig 7D). Compared to the control, the uNK cells-depleted mice exhibited reduced blood vessel densities (22%) and reduced numbers of large (>500 μm) VSF in the CTR (68%) at 8.5 dpc, although no significant differences in vascular densities and expressions of VEGFR2 and VEGFR3 were detected in the ULR and AMR (Fig 7D–F and Supporting Information Fig S6). These results indicated that uNK cells were involved in EEVSF.

### Dynamic postpartum changes in expressions of VEGF-A, PR and angiogenic growth factor receptors, and in mural cell coverage of uterine blood vessels

**Figure 8:**
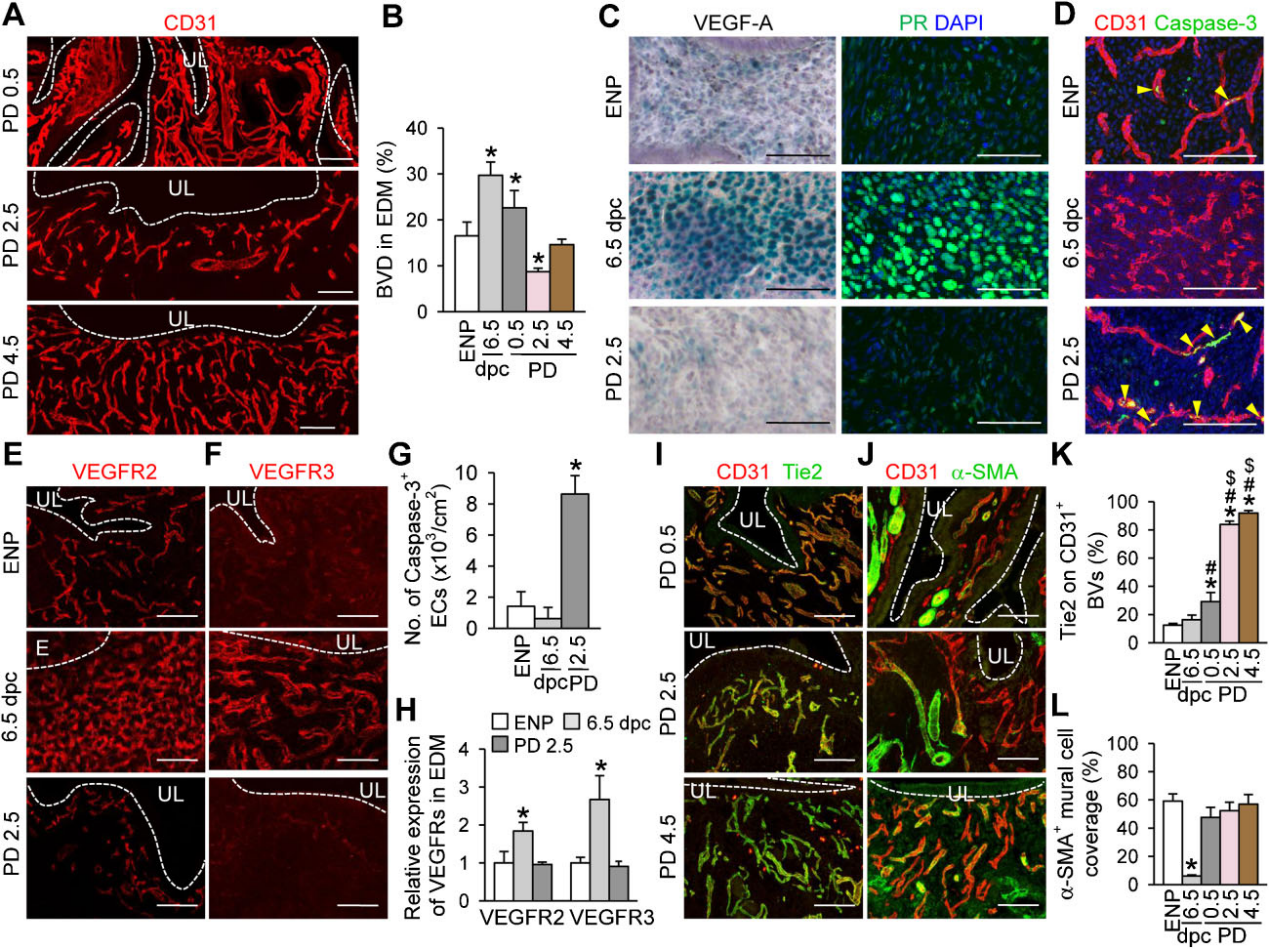
Dynamic changes of vascular density, expressions of VEGF-A, PR and angiogenic growth factor receptors, and mural cell coverage onto blood vessels in the postpartum uteri **A.** Images showing CD31^+^ BVs in the endometrium at PD 0.5, 2.5 and 4.5.**B, C.** Comparisons of CD31^+^ BVD (%) and expressions of VEGF-A and PR in the endometrium of ENP, 6.5 dpc, and PD 0.5, 2.5 and 4.5. Each group, *n* = 4 *p < 0.05 versus ENP by one-way ANOVA.**D–F** Images showing caspase-3^+^ ECs (yellow arrowheads) and expressions of VEGFR2 and VEGFR3 in the endometrium at ENP, 6.5 dpc and PD 2.5.**G, H.** Comparison of number of caspase-3^+^/CD31^+^ ECs and expressions of VEGFR2 and VEGFR3 (relative folds to the levels of ENP) in the endometrium in a given area (cm^2^) at ENP, 6.5 dpc and PD 2.5. Each group, *n* = 4 *p < 0.005 versus ENP by one-way ANOVA.**I, J.** Images showing Tie2 expressions and α-SMA^+^ coverage on the CD31^+^ BVs in the endometrium at PD 0.5, 2.5 and 4.5.**K, L.** Comparisons of Tie2 expression and α-SMA^+^ mural cell coverage on the CD31^+^ BVs (%) in the endometrium of ENP, 6.5 dpc, and PD 0.5, 2.5 and 4.5. Each group, *n* = 4 *p < 0.02 versus ENP; ^#^*p* < 0.04 versus 6.5 dpc; ^$^*p* < 0.0004 versus PD 0.5 by one-way ANOVA. UL, uterine lumen. All scale bars, 100 µm.

In contrast to in early pregnancy — after delivery, uterine sizes were sharply decreased and the endometrial blood vessels were regressed and maturated (Supporting Information Fig S7A and B) in parallel with sharp decreases in blood progesterone level (Nadal et al, 2009). The number of blood vessels in the endometrium was relatively high at PD 0.5, markedly reduced at PD 2.5, and rebounded at PD 4.5 up to the levels of ENP (Fig 8A and B). Concomitantly, VEGF-A and both forms of PR in the DSCs were sharply reduced in the postpartum uteri (Figs 4B and 8C). The number of caspase-3^+^ apoptotic ECs in the endometrium was markedly increased in PD 2.5 compared to ENP and 6.5 dpc (Fig 8D and G). Furthermore, expressions of VEGFR2 and VEGFR3 in the endometrial blood vessels were markedly reduced to levels similar to those of ENP (Fig 8E, F and H). In contrast, compared to 6.5 dpc, Tie2 expression and coverage of α-SMA^+^ mural cells on CD31^+^ blood vessels were 2.3- and 7.8-fold higher in PD 0.5, and gradually increased at PD 2.5 and 4.5 (Fig 8I–L). In particular, Tie2 expressions on blood vessels at PD 2.5 and 4.5 were ∼7.0-fold higher than that in the ENP (Fig 8K). These intriguing findings led us to examine the postpartum role of the Ang-Tie2 system in uteri. However, expressions of Ang1 and Ang2 were barely detectable in postpartum uteri (data not shown). Blockade of Ang-Tie2 signalling by administration of sTie2-Fc (25 mg/kg) at PD 0.5 and 1.5 did not induce any significant changes in vascular density, Tie2 expression, or coverage of α-SMA^+^ mural cells on CD31^+^ blood vessels at PD 2.5 in the treatment group compared with control Fc (Supporting Information Fig 7C–E). Thus, Tie2 may be involved in mural cell coverage and vessel regression and maturation in a ligand-independent manner in uteri during the postpartum period.

## DISCUSSION

**Figure 9:**
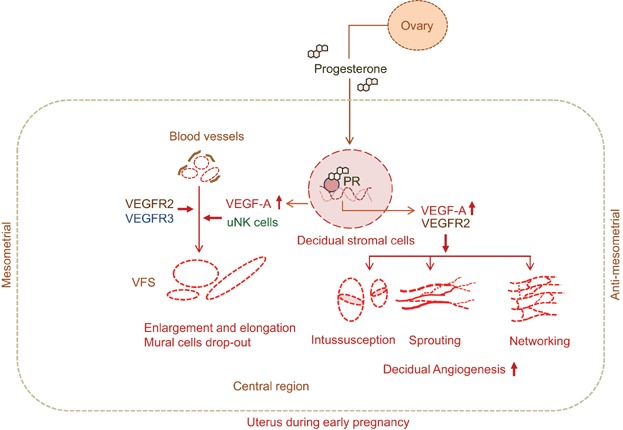
Scheme depicting dynamic and variable decidual angiogenesis (sprouting, intussusceptive and networking) and vascular remodelling (EEVSF and mural cell drop-out) and their regulations in the uterus of pregnant mouse during dpc 4.5–8.5.

In the present study, immunolocalization of blood vessels revealed that dynamic and variable decidual angiogenesis (including sprouting, intussusception and networking) and tremendous vascular remodelling (EEVSF and mural cell drop-out) occur distinctly at different regions in a temporal manner in the growing uterus during early pregnancy (Fig 9). In contrast, during the postpartum period, blood vessels in the endometrium rapidly regressed and maturated, with restoration of mural cell coverage. These observations led us to investigate the fine regulation of such angiogenesis and vascular remodelling by a key angiogenic growth factor system VEGF-VEGFR during these periods. Our spatiotemporal analyses indicated that decidual angiogenesis was mainly governed through P_4_-PR/VEGF-A-VEGFR2 signalling, but not through PlGF/VEGFR1 or Ang-Tie2 signalling, during early pregnancy (Fig 9). We further found that the P_4_-PR/VEGF-A-VEGFR2 signalling pathway, ligand-independent VEGFR3 signalling, and uNK cells positively regulate EEVSF (Fig 9). Disturbances of blood supply into the early pregnant uterus by impairments of these signalling pathways and cellular coordination could be associated with the first-trimester miscarriage, preeclampsia, placental failure and intrauterine growth restriction of births (Plaisier, 2011). Intriguingly, during the postpartum period, Tie2 signalling could be involved in vascular maturation at the endometrium in a ligand-independent manner. Postpartum hemorrhage can be caused by an impairment of the Tie2 signalling in the postpartum uterus (Oyelese & Ananth, 2010).

Although uterine blood vessels undertake the most dynamic vascular remodelling and angiogenesis during pregnancy, their spatiotemporal regulation within the uterus is poorly understood. In this study, we made several key findings and clarified their underlying mechanisms using genetically modified mice and pharmacological interventions. Firstly, our analyses revealed that VSF is a dilated arterio-venous vascular shunt, which is enlarged and elongated rapidly during the early pregnancy, and thus it has a bulk of blood flow for the growing uterus and embryo. VSF could be a transient blood reservoir for immediate need of blood to the growing embryo in emergency situations prior to placenta formation. Moreover, EEVSF could be a cue for spiral artery modification and placenta formation. Impairment of EEVSF could lead to trophoblast-independent preeclampsia, which produces hypertension and proteinuria during pregnancy, and even pregnancy failure in severe case (James et al, 2010; Pijnenborg et al, 2006; Plaisier, 2011). Secondly, among several angiogenic growth factors, we found that VEGF-A played a major role in decidual angiogenesis and a moderate role in EEVSF in the uterus during early pregnancy. Strikingly, VEGF-A was highly expressed in DSCs located in the AMR and the VSF area at CTR but not in the MR. Its expression coincided exactly with PR expression, but not with hypoxia or HIF expressions. At that time, the level of circulating P4 that is secreted mainly from ovary is rapidly increased (Nadal et al, 2009), and VEGFR2 is already highly expressed in the uterine blood vessels. Based on these findings, we propose that the P_4_-PR-VEGF-A axis mainly governs decidual angiogenesis and partially governs EEVSF. It remains to be elucidated which upstream regulator controls the expression of PR in DSCs that are located in the AMR and the VSF area at CTR. Our data suggest that putative vascular remodelling factors secreted from uNK cells distributed in the VSF region could play a coordinated role in EEVSF during early pregnancy. Consistent with a previous report (Plaks et al, 2008), our analyses of the temporal role of uDCs indicated that uDCs are crucial for embryo implantation and its associated vascular remodelling in the pregnant uteri during 3.5–5.5 dpc. However, we found that they are no longer crucial in the vascular remodelling including EEVSF in the pregnant uteri during 6.5–8.5 dpc after the successful embryo implantation.

Thirdly, during early pregnancy, VEGFR2 is highly expressed in most blood vessels regardless of the uterus region, while VEGFR3 is selectively and highly expressed in the ECs of VSF at the decidualization zone, and can be a biomarker of VSF. The VSF abruptly receives increased blood flow during early pregnancy, requiring a markedly increased mechanical stretching, which may induce VEGFR3 activation (Planas-Paz et al, 2012). Nevertheless, given that VEGF-C expression is relatively low or absent surrounding the VSF and that blockade of VEGF-C and VEGF-D did not change EEVSF, we propose that VEGFR3 could be involved in EEVSF in a mechano-transduction manner (Planas-Paz et al, 2012) and/or a ligand-independent manner, as has been observed in the blood vessels of growing retina (Benedito et al, 2012). Although the anti-VEGFR3 antibody did not disrupt pregnancy progression (Douglas et al, 2009), investigating the exact role of VEGFR3 in EEVSF will require further studies involving the genetic deletion of VEGFR3 or pharmacological inhibition of VEGFR3 kinase activity in the VSF.

Finally, our spatiotemporal analyses of the postpartum period uncovered several novel and intriguing findings on regression and maturation of blood vessel in the uterus. Blood vessels were pruned and regressed sharply with concomitant increase of apoptosis in endometrial ECs. These could be caused by sharp reductions of circulating P4 secreted from ovary (Nadal et al, 2009), expressions of PR and VEGF-A in the regressed DSCs, and expressions of VEGFR2 and VEGFR3 in the ECs. Intriguingly, the level of Tie2 expression and coverage of mural cells on the blood vessels were much higher than those in ENP and early pregnancy. Given that the levels of Ang1 and Ang2 were relatively low or absent around the Tie2-expressing blood vessels, and that sTie2 did not change the vascular remodelling, Tie2 could be involved in mural cell coverage and vascular regression and maturation in a ligand-independent manner in uteri during the postpartum period. To investigate the role of Tie2 in blood vessels during the postpartum period, further studies should involve genetic deletion of Tie2 in the blood vessels or pharmacological inhibition of Tie2 kinase activity.

In conclusion, our findings shed light on the previously undefined features and regulations of uterine angiogenesis and vascular remodelling during early pregnancy and the postpartum period. Dynamic but distinct decidual angiogenesis and vascular remodelling at different regions occur in the early pregnant uterus. In the rapidly growing uterus, P_4_-PR-regulated VEGF-A-VEGFR2 signalling is a key regulator for decidual angiogenesis, whereas P_4_-PR-regulated VEGF-A-VEGFR2 signalling, VEGFR3 signalling and uNK cells coordinately control EEVSF. In comparison, in the rapidly regressing uteri, Tie2 signalling could be involved in vascular maturation in a ligand-independent manner.

## MATERIALS AND METHODS

### Mice

Animal care and experimental procedures were performed under approval from the Animal Care Committees of KAIST and CHA University. Specific pathogen-free C57BL/6J, GFP^+^ (C57BL/6J), ROSA26-Cre^ERT2^ (C57BL/6J), CD11c:DTR (C57BL/6J). VEGFR1^TK−/−^ mice (Hiratsuka et al, 1998), VEGF-A^+/LacZ^ mice (Miquerol et al, 2000), VEGFR2^+/LacZ^ mice (Ema et al, 2006), VEGF-A^flox/flox^ mice (Gerber et al, 1999), PR-Cre mice (Soyal et al, 2005) and VEGF-C^+/LacZ^ mice (Karkkainen et al, 2004) were transferred and bred in our pathogen-free animal facilities. VEGF-A^+/LacZ^ mice (Miquerol et al, 2000) have elevated VEGF expression by 3′UTR insertion of the IRES-*lacZ*-pA sequence, resulting in increased stability of the VEGF-A transcripts, were used in this study to examine expression pattern of VEGF-A since they showed almost similar vascular structures and densities in the uterine endometrium of both pregnancy and non-pregnancy compared to those of wild-type mice. To deplete VEGF-A in PR^+^ DSCs in the uterus, VEGF-A^flox/flox^ mice were intercrossed with PR-Cre mice. To deplete DCs in a DTx-dependent manner, 100 ng of DTx (2 ng/g of body weight, Sigma–Aldrich) dissolved in PBS was injected into peritoneal cavity of CD11c:DTR mice at indicated days. Wild-type (designated as controls) mice were treated with DT in the same manner. Mice aged 7 to 8 weeks were used for this study unless otherwise specifically indicated. Females were mated with males, and the time that a vaginal plug was found was designated 0.5 dpc. In addition, the morning after birth was designated postpartum day (PD) 0.5. All mice were fed with *ad libitum* access to standard diet (PMI Lab diet) and water. All mice were anaesthetized by intramuscular injection of a combination of anaesthetics (80 mg/kg of ketamine and 12 mg/kg of xylazine) before being sacrificed.

### Preparations and treatments of reagents

To produce recombinant proteins- dimeric-Fc (Fc), VEGF-Trap and sVEGFR3-Fc (consisting with the first three immunoglobulin domains of human VEGFR3 and the Fc-domain of human antibody), stable CHO cell lines that secrete these recombinant proteins were used as previously described (Koh et al, 2010). Recombinant proteins in supernatant were purified by column chromatography with Protein A agarose gel (Oncogene) using acid elution. After purification, the recombinant proteins were quantified using the Bradford assay and confirmed by Coomassie blue staining after SDS-PAGE. Proteins were intraperitoneally injected by following dosages and schedules: VEGF-Trap (25 mg/kg) at 4.5, 5.5 or 6.5 dpc and sVEGFR3-Fc (25 mg/kg) at 6.5 and 7.5 dpc. As a control, Fc was injected in the same manner. P4 (Sigma–Aldrich) dissolved in peanut oil was intraperitoneally injected in 0.5 mg per mouse at 4.5 and 5.5 dpc. RU486 (8 mg/kg; Sigma–Aldrich) or ICI 182780 (1 mg/kg; Sigma–Aldrich) was intraperitoneally injected at 5.5 dpc. To eliminate uNK cells, anti-NKG2D antibody (7.5 mg/kg; MAB1547, R&D Systems) was intravenously injected through tail vein at 6.5 and 7.5 dpc (twice a day). As a control, Fc was injected in the same manner. All treated mice were sacrificed and examined at indicated days.

### Ovariectomy

To determine role of P4 upon exclusion of other ovarian hormones, a combination of ovariectomy and P4 replacement was performed according to procedures described previously (Douglas et al, 2009; Walter et al, 2005) with some modifications. Briefly, at 3.5 dpc, P4 (2.5 mg per mouse) was subcutaneously injected, and at 4 h later, the bilateral ovariectomy was performed. From the next day, the mice were treated with P4 (2.5 mg per mouse) twice a day in the same manner.

### Histological analyses

On the indicated days, the mice were anaesthetized by intramuscular injection of the combination of anaesthetics. Segments of the uterus containing implanted embryos were fixed in 1% paraformaldehyde, embedded with tissue freezing medium (Leica) or paraffin, and sectioned. Samples were blocked with 5% donkey (or goat) serum in PBST (0.03% Triton X-100 in PBS) and incubated for 3 h at room temperature (RT) with the following primary antibodies and lectin: anti-CD105 (rat, clone MJ7/18, eBioscience), anti-CD31 (hamster, clone 2H8, Millipore, Billerica, MA, USA), anti-PH3 (rabbit polyclonal, Millipore), anti-PR (rabbit polyclonal, Abcam), anti-caspase-3 (rabbit polyclonal, R&D Systems), FITC-conjugated anti-α-SMA (mouse, clone 1A4, Sigma–Aldrich), anti-RFP (rabbit polyclonal, Abcam), anti-VEGFR3 (goat polyclonal, R&D Systems), anti-VEGF-A (goat polyclonal, R&D Systems), anti-VEGFR2 (rabbit monoclonal, TO14, gifted by Dr. Rolf A. Brekken), anti-Prox-1 (rabbit polyclonal, Relia Tech GmbH), anti-CCR7 (rabbit polyclonal, Abcam), anti-Tie2 (rabbit polyclonal, Santa Cruz Biotechnology), anti-angiopoietin-1 (rabbit polyclonal, Ab Frontier), anti-Ang2 monoclonal (Aprogen) or biotin-conjugated anti-DBA lectin (Sigma–Aldrich). After several washes, the samples were incubated for 2 h at RT with the following secondary antibodies: Cy3-conjugated anti-hamster IgG (Jackson ImmunoResearch), Cy3- or FITC-conjugated anti-rabbit IgG (Jackson ImmunoResearch), Cy3-conjugated anti-rat IgG (Jackson ImmunoResearch), Cy3- or FITC-conjugated anti-goat IgG (Jackson ImmunoResearch) or PE-conjugated streptavidin (eBioscience). Nuclei were stained with 4′,6-diamidino-2-phenylindole (DAPI, Invitrogen). Then the samples were mounted in fluorescent mounting medium (DAKO) and immunofluorescent images were acquired using Zeiss LSM510 confocal fluorescence microscope (Carl Zeiss). To examine β-galactosidase activity, the cryo-sections were incubated with a staining solution [2 mM magnesium chloride, 5 mM potassium ferricyanide, 5 mM potassium ferrocyanide and 1 mg/mL 4-chloro-5-bromo-3-indolyl-β-d-galactopyranoside (X-gal) in PBS] at 37°C for 2–12 h. To detect the hypoxic area in the uterus, Hypoxyprobe-1™ (60 mg/kg, solid pimonidazole HCL; Natural Pharmacia International) was intravenously injected 45 min before perfusion-fixation. Then the uterus was harvested, sectioned and incubated with FITC-conjugated anti-hypoxyprobe antibody. To detect HIF-1α and HIF-2α, the antigens were retrieved in the paraffin-embedded sections by heating in a microwave for 5 min in sodium citrate solution (10 mM; pH 6.0). After blocking, the sections were incubated overnight at 4°C with anti-HIF-1α antibody (SC10790, Santa Cruz) and anti-HIF-2α antibody (NB100-122, Novus Biologicals). Then the sections were incubated with avidin–biotin–horseradish peroxidase (HRP) complex, and the signals were visualized with 3,3′-diaminobenzidine (DAB) immunostaining kit (K3468, DAKO) according to the manufacturer's instructions.

**The paper explained**

**PROBLEM:**

Profound neovascularization and marked vascular remodelling and regression take place in the uterus throughout and after pregnancy to control blood flow to the uterus as well as to the embryo. Impairments of these fundamental processes leads to a pregnancy failure; however, the features and regulation of these processes are poorly defined.

**RESULTS:**

Here we found that dynamic and variable decidual angiogenesis (sprouting, intussusception and networking), and active vigorous vascular remodelling such as enlargement and elongation of ‘vascular sinus folding’ (VSF) and mural cell drop-out occur distinctly in a spatiotemporal manner in the rapidly growing mouse uterus during early pregnancy — just after implantation but before placentation. Decidual angiogenesis is mainly regulated through VEGF-A secreted from the progesterone receptor (PR)-expressing decidual stromal cells, whereas P_4_-PR-regulated VEGF-A-VEGFR2 signalling, ligand-independent VEGFR3 signalling and uNK cells positively and coordinately regulate enlargement and elongation of VSF. During the postpartum period, Tie2 signalling could be involved in vascular maturation at the endometrium in a ligand-independent manner.

**IMPACT:**

Our findings indicate that two key vascular growth factor receptors — VEGFR2 and Tie2 — strikingly but differentially regulate decidual angiogenesis and vascular remodelling in rapidly growing and regressing uteri. For this reason, anti-angiogenic treatments affecting these key signalling pathways should be prohibited during pregnancy as well as postpartum period. In early pregnant uterus, proper enlargement and elongation of VSF could be important for avoiding a trophoblast-independent preeclampsia.

### Morphometric analyses

Morphometric measurements of blood vessel densities and sizes in the uteri were made on the sectioned tissues immunostained for CD31^+^ or CD105^+^ using a photographic analysis in ImageJ software (http://rsb.info.nih.gov/ij) or an LSM Image Browser (Carl Zeiss). For blood vessel density, CD31^+^ or CD105^+^ blood vessel area was measured in the ULR, CTR, AMR and total endometrium in each 0.53, 1.07, 1.56 and 3.16 mm^2^ area, and presented as percentage per the measured area. Size of blood vessel was defined as the longest diameter (μm) of each CD31^+^ or CD105^+^ blood vessel at the CTR in 1.07 mm^2^ area, and counted and subdivided by sizes. Numbers of vascular sprouts (>20 μm in length) were measured in the CTR, AMR and ENP in 3–4 random 0.42 mm^2^ areas. Numbers of intussusception (defined as a blood vessel having pillars in the lumen) were measured in 3–4 random 0.02 mm^2^ areas of the endometrium. Numbers of PH3^+^/CD31^+^ ECs or caspase-3^+^/CD31^+^ ECs were measured in 3 random 0.42 mm^2^ areas of the endometrium.

Expressions of VEGFR2 and VEGFR3 were determined by measurements of the signal densities in total endometrium of ENP, 6.5 dpc and PD 2.5 in each 1.59, 3.16 and 0.75 mm^2^ area, and presented as relative fold to the signal density of ENP that was regarded as 1. Coverage of α-SMC^+^ mural cells to blood vessels was calculated as α-SMC^+^ area divided by CD31^+^ blood vessel area in 4 random 0.42 mm^2^ areas of each endometrium, and presented as percentage. 2–3 pregnant uteri per each mouse were examined, and indicated number of mothers per each group was used for the analyses.

### Scanning electron microscopy

For vascular fixation, 8% paraformaldehyde was injected into left ventricle of the mice under the anaesthesia on indicated day. The uterine segments was fixed further with 8% paraformaldehyde for overnight, embedded with paraffin, and sectioned. The samples were freeze-dried in a lyophilizer for 24 h, and mounted on stubs and coated with ion by ion coater (KIC-1A, COXEM, Korea) operated at 6 mA for 60 s. Images were acquired using scanning electron microscope (SEM, S-4800, Hitachi, Japan) operated at 15 kV, 7 A.

### Primary culture of stromal cells

DSCs at 6.5 dpc, cardiac fibroblasts at P14, retinal astrocytes at P3 and MEFs at 13.5 dpc were isolated according to procedures described previously (Li et al, 2007; Scheef et al, 2005; Subramanian et al, 2002; Wernig et al, 2008). Briefly, each tissue was harvested, finely minced and digested with collagenase type II (1 mg/mL; Worthington), collagenase type IV (2 mg/mL; Worthington) and dispase II (0.02 mg/mL; Roche Applied Science) in DMEM for 1 h at 37°C. The digested cells were then plated onto type I collagen (BD Biosciences)-coated plate and were cultured in DMEM containing 10% fetal bovine serum (FBS), streptomycin (100 µg/mL) and penicillin (100 U/mL). The sub-cultured cells were harvested at 48 h after plating, and analyses for gene expressions were performed. To examine change of VEGF-A expression in primary cultured DSCs by P4, the cells were starved for 6 h, and then treated with P4 (10 µM) for indicated times.

### Semi-quantitative RT-PCR and quantitative real-time PCR

Total RNA was extracted from the uterus or the primary cultured stromal cells using TRIzol® Reagent (Invitrogen) according to the manufacturer's instructions. The RNA (2 µg) was reverse transcribed into cDNA using SuperScript II Reverse Transcriptase (Invitrogen). Semi-quantitative RT-PCR was performed using NobleZyme™ Tap (Noble Bio) with the indicated primers (see below). Quantitative real-time PCR was performed with the indicated primers using Bio-RadTM CFX96 Real-Time PCR Detection System (Bio-Rad). The real-time PCR data were analysed with Bio-Rad CFX Manager Software (Bio-Rad). Primers for the semi-quantitative RT-PCR (Semi) and quantitative real-time PCR are: *VEGFR2* (semi); forward 5′- ACCAGAAGTAAAAGTGATCCCAGA -3′ and reverse 5′- TCCACCAAAAGATGGAGATAATTT -3′, *VEGF-A* (semi); forward 5′- CAGGCTGCTCTAACGATGAA-3′ and reverse 5′- CAGGAATCCCAGAAACAACC-3′, *VEGF-A*; forward 5′- CAGAAGGAGAGCAGAAGTCC -3′ and reverse 5′- CTCCAGGGCTTCATCGTTA -3′, *VEGF-C*; forward 5′- CGTTCTCTGCCAGCAACATTACCAC -3′ and reverse 5′- CTTGTTGGGTCCACAGACATCATGG -3′, *PlGF*; forward 5′- CTGCTGGGAACAACTCAACAGAAGTG -3′ and reverse 5′- CTACAGCGACTCAGAAGGACACAGG -3′, *Ang1*; forward 5′- TAGAGCTACCAACAACAACAGCA -3′ and reverse 5′- CCCTTTAGCAAAACACCTTCTTT -3′, *Ang2*; forward 5′- CTGGTGAAGAGTCCAACTACAGG -3′ and reverse 5′- CGAATCCTTTGTGCTAAAATCAC -3′, *PDGF-A*; forward 5′- CTGCTCCTCGGCTGCGGATACCTC -3′ and reverse 5′- GAGTCGCTGGAGGTCCCGGATGCTG -3′, *TGF-β1*; forward 5′- CAAGGAGACGGAATACAGGGCTTTC -3′ and reverse 5′- GTTCATGTCATGGATGGTGCCCAG -3′, *GAPDH*; forward 5′- GTCGTGGAGTCTACTGGTGTCTTCAC -3′ and reverse 5′- GTTGTCATATTTCTCGTGGTTCACACCC -3′.

### Western blotting

On the indicated days, the uterus, mammary gland, ovary, brain and kidney were homogenized in ice-cold buffer containing a protease inhibitor cocktail. Each protein was separated by SDS-PAGE gel and transferred to PVDF membranes. After blocking with 5% skim milk, the membranes were incubated with anti-PR antibody (rabbit polyclonal, Abcam) and anti-Actin antibody (rabbit polyclonal, Sigma–Aldrich) in blocking buffer overnight at 4°C and then with hrP-conjugated secondary antibody for 2 h at RT. Signals were developed with enhanced chemiluminescence hrP substrate (Millipore) and detected using LAS-1000 mini (Fuji film).

### Flow cytometry

Segments of the uterus containing implanted embryos were harvested. After removal of embryonic tissues, samples were finely minced and digested with collagenase type II (1 mg/mL), collagenase type IV (2 mg/mL) and dispase II (0.02 mg/mL) for 1 h at 37°C. The digested cells were resuspended in HBSS/2% FBS at 1 × 10^6^ cells per 100 µL. The cells were incubated for 20 min with following antibodies: PE-conjugated anti-mouse CD31 (clone 390, eBioscience) and FITC-conjugated anti-mouse CD45 (eBioscience). After washing in HBSS/2% FBS twice, the cells were analysed by FACS Aria II (Beckton Dickinson). Dead cells were excluded by 7-aminoactinomycin D (7-AAD, Invitrogen). Data were analysed using FlowJo software (Tree Star Inc.).

### Statistical analysis

Values are presented as mean standard deviation (SD). Significant differences between means were determined by unpaired Student *t* test or analysis of variance with one-way ANOVA followed by the Student-Newman-Keuls test. Statistical significance was set at *p* < 0.01 or *p* < 0.05.

## Supplementary material

As a service to our authors and readers, this journal provides supporting information supplied by the authors. Such materials are peer reviewed and may be re-organized for online delivery, but are not copy-edited or typeset. Technical support issues arising from supporting information (other than missing files) should be addressed to the authors.
