# Supplementary material for: VEGF-A regulated by progesterone governs uterine angiogenesis and vascular remodelling during pregnancy
Source: EMBO Mol Med. 2013 Aug 2;5(9):1415–30. doi: 10.1002/emmm.201302618 (PMC3799495; doi:10.1002/emmm.201302618)
Supplement: Supplementary file 1 [file emmm0005-1415-SD1.pdf]

EMBO Molecular Medicine

VEGF-A regulated by progesterone governs uterine angiogenesis and vascular remodeling during pregnancy

Minah Kim, Mr. Hyeung Ju Park, Jae Won Seol, Jeon Yeob Jang, Young-Suk Cho, Kyu Rae Kim, Youngsok Choi, John P. Lydon, Francesco J. DeMayo, Masabumi Shibuya, Napoleone Ferrara, Hoon-Ki Sung, Andras Nagy, Kari Alitalo and Gou Young Koh

Corresponding author: Gou Young Koh, KAIST

Review timeline:

Submission date:	08 February 2013
Editorial Decision:	13 March 2013
Revision received:	28 May 2013
Editorial Decision:	12 June 2013
Revision received:	15 June 2013
Editorial Decision:	20 June 2013
Revision received:	24 June 2013
Accepted:	25 June 2013

Transaction Report:

(Note: With the exception of the correction of typographical or spelling errors that could be a source of ambiguity, letters and reports are not edited. The original formatting of letters and referee reports may not be reflected in this compilation.)

Editor: Roberto Buccione

1st Editorial Decision 13 March 2013

Thank you for the submission of your manuscript to EMBO Molecular Medicine. We have now received reports from the three Reviewers whom we asked to evaluate your manuscript.

You will see that while the two Reviewers are generally supportive, one Reviewer is quite negative.

Reviewer 1, while acknowledging the interest and quality of your work, is of the opinion that the manuscript bears limited clinical relevance and thus ill-suited to this journal. At this point, this will not be a basis for rejection provided you address this issue in your manuscript by discussing throughout how your findings are not only relevant for physiology but also in pathological conditions.

Reviewer 2 notes that the text should be made more accessible for a general readership. I very much agree. S/he also requires important clarifications on data interpretation that require careful, complete consideration.

Reviewer 3 is concerned that some claims as mentioned in the Discussion are not sufficiently supported by appropriate experimentation and/or quantification. S/he also mentions other items that require your action.

While publication of the paper cannot be considered at this stage, we would be prepared to consider a suitably revised submission, with the understanding that the Reviewers' concerns must be fully addressed with additional experimental data where appropriate and that acceptance of the manuscript might entail a second round of review.

Please note that it is EMBO Molecular Medicine policy to allow a single round of revision only and that, therefore, acceptance or rejection of the manuscript will depend on the completeness of your responses included in the next, final version of the manuscript.

As you know, EMBO Molecular Medicine has a "scooping protection" policy, whereby similar findings that are published by others during review or revision are not a criterion for rejection. However, I do ask you to get in touch with us after three months if you have not completed your revision, to update us on the status. Please also contact us as soon as possible if similar work is published elsewhere.

I look forward to seeing a revised form of your manuscript as soon as possible.

***** Reviewer's comments *****

Referee #1 (Comments on Novelty/Model System):

Dear colleague,
thank you very much for the possibility to read this exciting work.

This is a very interesting model in terms of how uterine vascularisation in mouse pregnancy might be regulated by TIE2 and VEGFR2.
The presented manuscript tries to conclude that VEGFR2 and TIE2 could be significant contributors to vasculopathy in the decidua and cause trophblast dependent preeclampsia.
Although the results are striking the relevance to human disease are very limited for the following reasons.
a. mice do not develop preeclampsia, if at all they develop preeclamptic phenotypes. Endothelial dysfunction is one of the characteristics.
b. it is well known that anti angiogenic factors / therapies (anti VEGF antibodies etc) cause preeclamptic like phenotypes due to its impact on vascular function.
As a logical consequence such a therapy would not be used in a pregnancy condition to begin with.
This work though is a very important addition to our knowledge on uterine vascularisation but yet shows only little relevance to a pathological condition.
The reviewer is aware that this model is 'as good as it gets' since studies in the maternal decidua are very difficult.
I suggest the authors submit this work to a more suited journal such as Development.

Referee #1 (Remarks):

This is a well designed study. The study as is lacks the clinical relevance and should be submitted to a different journal.

Referee #2 (Remarks):

The study by Kim et al is a high quality contribution from experts in the field, showing the role of progesterone in inducing VEGFA expression during pregnancy in the mouse. The authors show that VEGFA is a critical factor in the dynamic regulation of the uterine vasculature and that VEGFA is induced by progesterone in a hypoxia-independent manner. The data are generated using high quality models and stringently evaluated. The expression patterns and ligand-responsiveness of VEGFR2 and VEGFR3 are to some extent unexpected and require further studies.
1. The text would be more readable with fewer abbreviations. This is important in a general journal. The authors also use some non-standard abbreviations such as BV for blood vessels and BVD for blood vessel density (neither of which need to be abbreviated) and NKC instead of NK cells. Certain sentences have as many as six abbreviations. I suggest that the authors go through the text to make it more accessible.

2. In Fig. 1, the authors imply intussusception as a mechanism for the rapid expansion of the vascular bed. This is likely to be the case, but difficult to discern from the figure (D and H) and also not essential for the study.
3. The expression pattern of VEGFR2 (Fig. 3C) overlaps with that of VEGFA in the secondary decidual zone, but not in other regions in the pregnant uterus. Is this due to the layer examined (i.e., when sectioning through the uterus, would there be coexpression in the rostral/caudal orientation rather than the dorsal/ventral (which is shown now)?
4. In Figs. 6 and 8, the authors study expression of VEGFR3 in the uterus. It is unclear whether VEGFR3 is expressed in blood vessels, lymphatic vessels or both? Is VEGFR3 coexpressed with VEGFR2? Does VEGFR3 expression decrease as a consequence of the VEGFtrap treatment?
5. In Fig. 7, showing effect of NK cell neutralization, the authors have immunostained for VEGFR3. What is the pattern for VEGFR2?
6. The a-SMA expression shown in Fig. 8, depicts strong staining on structures that do not seem to be CD31-positive. It may be preferable to use another marker for mural cells.
7. Tie2, which is induced after birth, as shown in panels I and K in Fig. 8, is interpreted as active in a ligand-independent manner. This is possible but would need to be consolidated. Does turnover of GFP play a role in the interpretation? What about expression of Tie2 ligands?

Referee #3 (Comments on Novelty/Model System):

Some of the claims are not substantiated by quantitative analysis.

Referee #3 (Remarks):

1. Page 9 'we compared relative expression levels of the growth factors in primary cultured DSCs to those of other primary cultured stromal cells, such as cardiac fibroblasts, retinal astrocytes, and mouse embryonic fibroblasts (MEFs). ' How does that reflect the true expression in situ.
2. The stroma cell composition for the different primary cultures can be very different
Obviously expression in culture can be very different from the endogenous
The post partum data is not relevant or connected to the rest of the paper.
3. Discussion beginning ' sprouting, intussusception, and networking)' is that demonstrated experimentally and quantified? No methods are listed in the morphometric analysis and no quantitative results are provided.
4. Page 17 'Consistent with a previous report (Plaks et al, 2008), our analyses of the temporal role of uDCs indicated that uDCs are crucial for embryo implantation, rather than that the vascular remodeling occurred after successful embryo implantation. ' appears to be an incorrect citation, as that paper actually showed a role for DCs in decidua associated vascular maturation.
5. The post partum data is not connected to the main story of this manuscript

1st Revision - authors' response 28 May 2013

Answers to the editor's and reviewers' comments

We deeply appreciate the editor and reviewers for their thoughtful and critical comments, which have undoubtedly provided us with valuable opportunities to improve our work. We have performed additional experiments and have revised the manuscript, following the advice provided, to address the issues raised by the reviewers.

The Editor

Reviewer 1, while acknowledging the interest and quality of your work, is of the opinion that the manuscript bears limited clinical relevance and thus illsuited to this journal. At this point, this will not be a basis for rejection provided you address this issue in your manuscript by discussing throughout

© EMBO

how your findings are not only relevant for physiology but also in pathological conditions.

We have now included additional comments in the revised Discussion section to emphasize that our findings are not only relevant for physiology but also in pathological conditions, in accord with the focus of EMBO Molecular Medicine.

Reviewer 2 notes that the text should be made more accessible for a general readership. I very much agree. S/he also requires important clarifications on data interpretation that require careful, complete consideration.

As the reviewer indicated, we have avoided non-standard abbreviations as much as possible, and have made the revised manuscript to be more readable.

Reviewer 3 is concerned that some claims as mentioned in the Discussion are not sufficiently supported by appropriate experimentation and/or quantification. S/he also mentions other items that require your action.

While we appreciate the reviewer's comments and have considered them thoroughly, we disagree with most of the comments raised by the reviewer. Nevertheless, we have tried to address the comments as much as we could.

Referee #1

(Comments on Novelty/Model System): Dear colleague, thank you very much for the possibility to read this exciting work. This is a very interesting model in terms of how uterine vascularisation in mouse pregnancy might be regulated by TIE2 and VEGFR2. The presented manuscript tries to conclude that VEGFR2 and TIE2 could be significant contributors to vasculopathy in the decidua and cause trophblast dependent preeclampsia. Although the results are striking the relevance to human disease are very limited for the following reasons.

a. mice do not develop preeclampsia, if at all they develop preeclamptic phenotypes. Endothelial dysfunction is one of the characteristics.

b. it is well known that anti angiogenic factors / therapies (anti VEGF antibodies etc) cause preeclamptic like phenotypes due to its impact on vascular function. As a logical consequence such a therapy would not be used in a pregnancy condition to begin with. This work though is a very important addition to our knowledge on uterine vascularisation but yet shows only little relevance to a pathological condition. The reviewer is aware that this model is 'as good as it gets' since studies in the maternal decidua are very difficult. I suggest the authors submit this work to a more suited journal such as Development.
(Remarks): This is a well designed study. The study as is lacks the clinical relevance and should be submitted to a different journal.

We are delighted by this favorable comment and encouragement. In response to the reviewer's comment, we have included additional comments in the revised Discussion section to emphasize that our findings are not only relevant for physiology but also in pathological conditions, in accord with the focus of EMBO Molecular Medicine.
Disturbances of blood supply into the early pregnant uterus by impairments of these signaling pathways and cellular coordination could be associated with the firsttrimester miscarriage, preeclampsia, placental failure, and intrauterine growth restriction of births (Plaisier M, 2011).
Postpartum hemorrhage can be caused by an impairment of the Tie2 signaling in the postpartum uterus (Oyelese Y and Ananth CV, 2010).

Referee #2 (Remarks):

The study by Kim et al is a high quality contribution from experts in the field, showing the role of progesterone in inducing VEGFA expression during pregnancy in the mouse. The authors show that VEGFA is a critical factor in the dynamic regulation of the uterine vasculature and that VEGFA is induced by progesterone in a hypoxia-independent manner. The data are generated using high quality models and stringently evaluated. The expression patterns and ligand-responsiveness of VEGFR2 and VEGFR3 are to some extent unexpected and require further studies.
1. The text would be more readable with fewer abbreviations. This is important in a general journal. The authors also use some non-standard abbreviations such as BV for blood vessels and BVD for blood vessel density (neither of which need to be abbreviated) and NKC instead of NK cells. Certain sentences have as many as six abbreviations. I suggest that the authors go through the text to make it more accessible.

As the reviewer indicated, we have avoided non-standard abbreviations as much as possible, and have made the revised manuscript to be more readable.

2. In Fig. 1, the authors imply intussusception as a mechanism for the rapid expansion of the vascular bed. This is likely to be the case, but difficult to discern from the figure (D and H) and also not essential for the study.

This is a valid point, but we would like to explain our findings in detail to the reviewer. As we described in the original manuscript, our detailed immunovisualization of CD105 (endoglin) or CD31 in the mid-sectioned uteri revealed that a putative intussusceptive pillar (Figure 1D) was frequently detected in the central region of endometrium at 4.5 and 6.5 dpc, whereas it was less frequently detected in other regions of pregnant uteri. We confirmed the intussusception at the electron microscopic level by showing interposed ECs as pillars inside of the vascular lumen (Figure 1H). We have included the finding obtained by scanning electron microscopy to discern the intussusception from other vascular remodeling processes in the revised supplementary figures.
Therefore, we believe that it is discernible from other vascular remodeling processes, and its description could be important in furthering our understanding of how uterine vasculatures are newly formed during early pregnancy.

3. The expression pattern of VEGFR2 (Fig. 3C) overlaps with that of VEGFA in the secondary decidual zone, but not in other regions in the pregnant uterus. Is this due to the layer examined (i.e., when sectioning through the uterus, would there be coexpression in the rostral/caudal orientation rather than the dorsal/ventral (which is shown now)?

This is an enlightening point that needs to be confirmed. We performed additional experiment to examine the expression patterns of VEGF-A and VEGFR2 in the sections made through the rostral/caudal orientation. The findings were consistent with the findings made through the dorsal/ventral orientation.

4. In Figs. 6 and 8, the authors study expression of VEGFR3 in the uterus. It is unclear whether VEGFR3 is expressed in blood vessels, lymphatic vessels or both? Is VEGFR3 coexpressed with VEGFR2? Does VEGFR3 expression decrease as a consequence of the VEGFtrap treatment?

We appreciate these critical comments. In response to the comment regarding the co-expression, we have included the additional result and its description in the revised manuscript as following.
VEGFR3, the cognate receptor of VEGF-C/D, was strongly expressed, together with VEGFR2, in the ECs of the vascular sinus folding (VSF) and the lymphatic vessels of early pregnant uteri (Figure 6A and Supplementary Figure 5).

In response to the comment regarding the change of VEGFR3 expression as a consequence of the VEGF-Trap treatment, we performed additional experiments. As a result, because the VEFG-Trap treatment markedly decreased number of largesized (>500 _m) VSF and VEGFR3 is mainly expressed in the large-sized VSF, VEGFR3 expression was largely decreased in the blood vessel of early pregnant uteri by the VEGF-Trap treatment. Since this result is not critical for this study, we show this result only to the reviewer (Figure R1 for the reviewer 2, please see page 7 (figure removed from PRPF)) and have not included it in the revised manuscript.

5. In Fig. 7, showing effect of NK cell neutralization, the authors have immunostained for VEGFR3. What is the pattern for VEGFR2?

We performed additional experiments to examine the pattern of VEGFR2, and have now included the data and their descriptions in the revised manuscript.
Compared to the control, the uNK cells-depleted mice exhibited reduced blood vessel densities (22%) and reduced numbers of large (>500 _m) VSF in the CTR (68%) at 8.5 dpc, although no significant differences in vascular densities and expressions of VEGFR2 and VEGFR3 were detected in the ULR and AMR (Figure 7D–F and Supplementary Figure 7).

6. The a-SMA expression shown in Fig. 8, depicts strong staining on structures that do not seem to be CD31-positive. It may be preferable to use another marker for mural cells.

Because the signal of _-SMA is too high, it sometimes masks the signal of CD31+ ECs in the merged images. In addition, the _-SMA+/CD31- blood vessels (shown at PD4.5 in Figure 8J) could be regressing blood vessels in the endometrium during the postpartum period. We excluded the _-SMA+/CD31- blood vessels for the analysis in the original manuscript.
As the reviewer suggested, we additionally examined the coverage of PDGFR-_+ pericytes on the CD31+ blood vessels in the endometrium during the postpartum periods. The finding of the coverage of PDGFR-_+ pericytes is consistent with the finding of the coverage of _-SMA+ mural cells. Given that the manuscript has already reached the space limitation and this could be another supporting result, we show this result only to the reviewer (Figure R2A and B for the reviewer 2, page 8 (figure removed from PRPF)), and have not included it in the revised manuscript.

7. Tie2, which is induced after birth, as shown in panels I and K in Fig. 8, is interpreted as active in a ligand-independent manner. This is possible but would need to be consolidated. Does turnover of GFP play a role in the interpretation? What about expression of Tie2 ligands?

This is an enlightening point that we need to address accurately. We performed additional experiments for Tie2 immuno-staining in the postpartum uteri, and found that the result of Tie2 immuno-staining was consistent with the result of Tie2-GFP signal. We have replaced the result of Tie2 immuno-staining with the result of Tie2-GFP signal in the revised manuscript to avoid possible misinterpretation, as the reviewer suggested.
As we described in the original manuscript, we found that rare expressions of Ang1 and Ang2 were detected in the postpartum uteri by immunohistochemical analysis and by using respective gene-reporter mouse models. We show a part of the results only to the reviewer (Figure R2C for the reviewer 2, page 8 (figure removed from PRPF)), and have not included them in the revised manuscript because it will exceed the space limitation and we believe that it is not pivotal to this study.

Referee #3 (Comments on Novelty/Model System):
Some of the claims are not substantiated by quantitative analysis.

We have now included quantitative analyses of the data in the revised manuscript.

Referee #3 (Remarks):

1. Page 9 'we compared relative expression levels of the growth factors in primary cultured DSCs to those of other primary cultured stromal cells, such as cardiac fibroblasts, retinal astrocytes, and mouse embryonic fibroblasts (MEFs). ' How does that reflect the true expression in situ.

We respect the reviewer's opinion but would still like to further explain our point of view on this experiment. By showing this result, we wanted to address the higher expression of VEGF-A in the DSCs, particularly when compared to the levels in other stromal cells. In fact, quantitative real-time PCR analyses on expressions of VEGF-A, VEGF-C and Ang2 in the primary stromal cells were well matched with the data obtained and analyzed using VEGF-A, VEGF-C reporter mouse models and immuno-staining of Ang2. Moreover, previous studies indicate that, in addition to placental trophoblasts, PIGF is highly expressed in the decidual stromal cells compared to other stromal cells (Ghosh et al., Molecular Human Reproduction 6:935-041, 2000; De Falco S Experimental Molecular Medicine 44:1-9, 2012). Therefore, we believe that the relative expression levels of the growth factors in primary cultured DSCs to those of other primary cultured stromal cells were faithfully able to reflect the true expressions *in situ*.

2. The stroma cell composition for the different primary cultures can be very different. Obviously expression in culture can be very different from the endogenous.

We admit that the stromal cell composition for the different primary cultures can be very different. Therefore, as we explained in the Materials and Methods, the primary cells were isolated by the same procedure and incubated under the same conditions to minimize a possible difference in stromal cell composition. In any event, as we responded to Comment 1, the data obtained from the primary cultured stromal cells faithfully reflected the true expressions *in situ*.

3. Discussion beginning ' sprouting, intussusception, and networking)' is that demonstrated experimentally and quantified? No methods are listed in the morphometric analysis and no quantitative results are provided.

In response to this comment, we performed additional analyses, and we have now provided the methods, morphometric analyses and quantitative results in the revised manuscript accordingly.

4. Page 17 'Consistent with a previous report (Plaks et al, 2008), our analyses of the temporal role of uDCs indicated that uDCs are crucial for embryo implantation, rather than that the vascular remodeling occurred after successful embryo implantation. ' appears to be an incorrect citation, as that paper actually showed a role for DCs in decidua associated vascular maturation.

As the reviewer indicated, we made an error in citation. We have now replaced it with the correct reference (Jung et al., 2004) in the revised manuscript.
As we described in the Results section (page 14) and Table S1, uDCs play a critical role in establishing embryo implantation, but not in uterine vascular remodeling occurring after successful embryo implantation, which is inconsistent with a previous report (Plaks et al, 2008). The reason for this difference remains to be investigated in the future as a separate study because it is beyond the scope of this study.

5. The post partum data is not connected to the main story of this manuscript. The post partum data is not relevant or connected to the rest of the paper.

We believe that it would be insightful and informative if we compare the key structural features and their molecular regulations of angiogenesis and vascular

remodeling in the rapidly growing uteri with those in the rapidly regressing uteri. In fact, we found several key events in the uteri during postpartum, which are reciprocally connected with the events that occur in the uteri during early pregnancy. Therefore, we would like to retain the data instead of completely deleting it.

2nd Editorial Decision 12 June 2013

Thank you for the submission of your revised manuscript to EMBO Molecular Medicine.

We have now heard back from the three Reviewers whom we asked to re-evaluate your revised manuscript.

You will see that two Reviewers have remaining issues, some of which prevent us from considering publication at this time.

Reviewer 1 remains unconvinced of the relevance to human pathology. As mentioned previously, this will not be a basis for rejection but I do urge you to take his/her new comments into consideration.

Reviewer 3 while acknowledging your replies to his/her previous comments, has some remaining concerns, chiefly concerning the method used for quantitative morphometric analysis, which should be adequately described and supported. Reviewer 3 also notes two other items that require your action.

As you know, we would normally not allow a second revision. I am prepared in this case, however, to give you another opportunity to improve your manuscript, with the understanding that the Reviewers' concerns must be fully addressed with additional experimental data where appropriate and that next version of the manuscript will undergo a third and final round of review with Reviewer 3.

I look forward to seeing a revised form of your manuscript as soon as possible.

***** Reviewer's comments *****

Referee #1 (Comments on Novelty/Model System):

The model system is interesting but confirmation in human specimen are still lacking. Defects in maternal spiral artery vascular function / remodeling can contribute to disease resulting in IUGR and preeclampsia, if the authors claim that hypoxia is the only cause of preeclampsia. (Which is likely not the only cause since we are dealing with a syndrome). Unfortunately this study is still limited to rodents and the authors did yet not convincingly provide evidence to human pathology.

Referee #1 (Remarks):

Unfortunately the authors were unable to convince me about the relevance to human pathology since experimental evidence is lacking. The mechanism in rodents is nicely presented but the application to human is still speculative.

Referee #2 (Comments on Novelty/Model System):

The authors have undertaken an ambitious revision and responded carefully to my comments. Clearly, the quality and novelty of this contribution are very high and the study is mature. The medical impact may not be as high considering the species differences. However, mouse is obviously the most relevant available model.

Referee #2 (Remarks):

The authors have undertaken an ambitious revision and responded adequately to my criticisms.

Referee #3 (Comments on Novelty/Model System):

I appreciate the addition of quantitative morphometric analysis. However the method is not adequately described. 3-4 regions is not many. Was this done on one section only? How many implantationr sites were measured per group? How many different mothers?

Referee #3 (Remarks):

Introduction, page 6 last line, 'normalization' of vessels is borrowed from the cancer field, but is out of context here in a process which is entirely 'normal'. The authors should consider a more specific tern, ie vascular maturation.

The reference for Plaks et allon the role of DCs in the decidua was replaced with Jung et al (listed as 2004 in the response but as 2004in the manuscript). the paper now cited is : In vivo depletion of CD11c+ dendritic cells abrogates priming of CD8+ T cells by exogenous cell-associated antigens. This manuscript describes the role of DCs in Malarian and Listeria and other for the animal model is not relevant. The citation within the manuscript again makes false claims for the paper cited. Please review all citations for accuracy of the claims made.

2nd Revision - authors' response 15 June 2013

Answers to the editor's and reviewers' comments

We deeply appreciate the editor and reviewers for their thoughtful, favorable and constructive comments, which have undoubtedly provided us with valuable opportunities to improve our work. We now have addressed the issues raised by the reviewer 3 accordingly.

The Editor

Reviewer 1 remains unconvinced of the relevance to human pathology. As mentioned previously, this will not be a basis for rejection but I do urge you to take his/her new comments into consideration.

As we addressed in the revised Discussion, our study particularly provides new insights to find causes of human first-trimester miscarriage, preeclampsia, placental failure, and intrauterine growth restriction of births that are resulted from unknown causes, and are difficult to study in human. Because the mouse system provides a reliable translation to human pathology in many aspects, our study would be largely applicable to understand the roles of angiogenesis and vascular remodeling in human pregnancy pathophysiology, which needs to be explored in the future. In this context, it would be appreciated if the comment raised by the reviewer 1 is no longer subjected to consideration.

Reviewer 3 while acknowledging your replies to his/her previous comments, has some remaining concerns, chiefly concerning the method used for quantitative morphometric analysis, which should be adequately described and supported. Reviewer 3 also notes two other items that require your action.

We revised the manuscript accordingly

Referee #3 (Comments on Novelty/Model System):

I appreciate the addition of quantitative morphometric analysis. However the method is not adequately described. 3-4 regions is not many. Was this done on one section only? How many implantation sites were measured per group? How many different mothers?

We believe that counting the numbers of vascular sprouts and intussusception in 3-4 random regions per each mid-section of the pregnant uterus is adequate to represent the findings. We examined 2-3 pregnant uteri per each mouse, and indicated number of mothers was analyzed for each group. However, as the reviewer indicated, we carelessly omitted the number of sample sizes in the revised Figure 1. We have now included the numbers of the mothers examined for these analyses in the re-revised Figure legend accordingly.

Referee #3 (Remarks):

Introduction, page 6 last line, 'normalization' of vessels is borrowed from the cancer field, but is out of context here in a process which is entirely 'normal'. The authors should consider a more specific tern, ie vascular maturation.

We agree to the reviewer's comment. We have changed the word throughout the text.

...Moreover, when we determined how blood vessels are regressed and undergoing vascular maturation in the endometrium during the postpartum period, ...

The reference for Plaks et allon the role of DCs in the decidua was replaced with Jung et al (listed as 2004 in the response but as 2004in the manuscript). the paper now cited is : In vivo depletion of CD11c+ dendritic cells abrogates priming of CD8+ T cells by exogenous cell-associated antigens. This manuscript describes the role of DCs in Malarian and Listeria and other for the animal model is not relevant. The citation within the manuscript again makes false claims for the paper cited. Please review all citations for accuracy of the claims made.

As the reviewer indicated, we made an error in the citation in the revised Results and Discussion. We have now replaced it with the paper published by Plaks et al (2008), which had been cited in the original manuscript, and rephrased the sentence accordingly. We rechecked the rest of references to confirm the claims accordingly.

... Consistent with a previous report (Plaks et al, 2008), our analyses of the temporal role of uDCs indicated that uDCs are crucial for embryo implantation and its associated vascular remodeling in the pregnant uteri during 3.5-5.5 dpc. However, we found that they are no longer crucial in the vascular remodeling including EEVSF in the pregnant uteri during 6.5-8.5 dpc after the successful embryo implantation. ..

3rd Editorial Decision 20 June 2013

Thank you for the submission of your revised manuscript to EMBO Molecular Medicine. I am pleased to inform you that we will be able to accept your manuscript pending the following final amendments:

1) As per our Author Guidelines, the description of all reported data that includes statistical testing must state the name of the statistical test used to generate error bars and P values, the number (n) of independent experiments underlying each data point (not replicate measures of one sample), and the actual P value for each test (not merely 'significant' or 'P < 0.05').

2) Please submit the final version of the revised manuscript without visible corrections and amendements (i.e. blue text).

Please submit your revised manuscript within two weeks. I look forward to seeing a revised form of your manuscript as soon as possible.

© EMBO

3rd Revision - authors' response 24 June 2013

We are delighted to hear that our manuscript entitled “VEGF-A regulated by progesterone governs uterine angiogenesis and vascular remodeling during pregnancy (EMM-2013-02618-V4)” is acceptable upon minor corrections.

We have revised our manuscript following your suggestions. We now have included the name of statistical tests used to generate error bars and P values, the number of independent experiments, and the actual P values for each test.
