# Supplementary material for: VEGF-A regulated by progesterone governs uterine angiogenesis and vascular remodelling during pregnancy
Source: EMBO Mol Med. 2013 Aug 2;5(9):1415–30. doi: 10.1002/emmm.201302618 (PMC3799495; doi:10.1002/emmm.201302618)
Supplement: Supplementary file 2 [file emmm0005-1415-SD2.pdf]

EMM-2013-02618

Supplementary Information

VEGF-A Regulated by Progesterone Governs Uterine Angiogenesis and Vascular Remodeling during Pregnancy

Minah Kim, Hyeung Ju Park, Jae Won Seol, Jeon Yeob Jang, Young-Suk Cho,
Kyu Rae Kim, Youngsok Choi, John P. Lydon, Francesco J. DeMayo, Masabumi Shibuya,
Napoleone Ferrara, Hoon-Ki Sung, Andras Nagy, Kari Alitalo, Gou Young Koh

Contents

1. Supplementary Figure 1
2. Supplementary Figure 2
3. Supplementary Figure 3
4. Supplementary Figure 4
5. Supplementary Figure 5
6. Supplementary Figure 6
7. Supplementary Figure 7
8. Supplementary Table 1

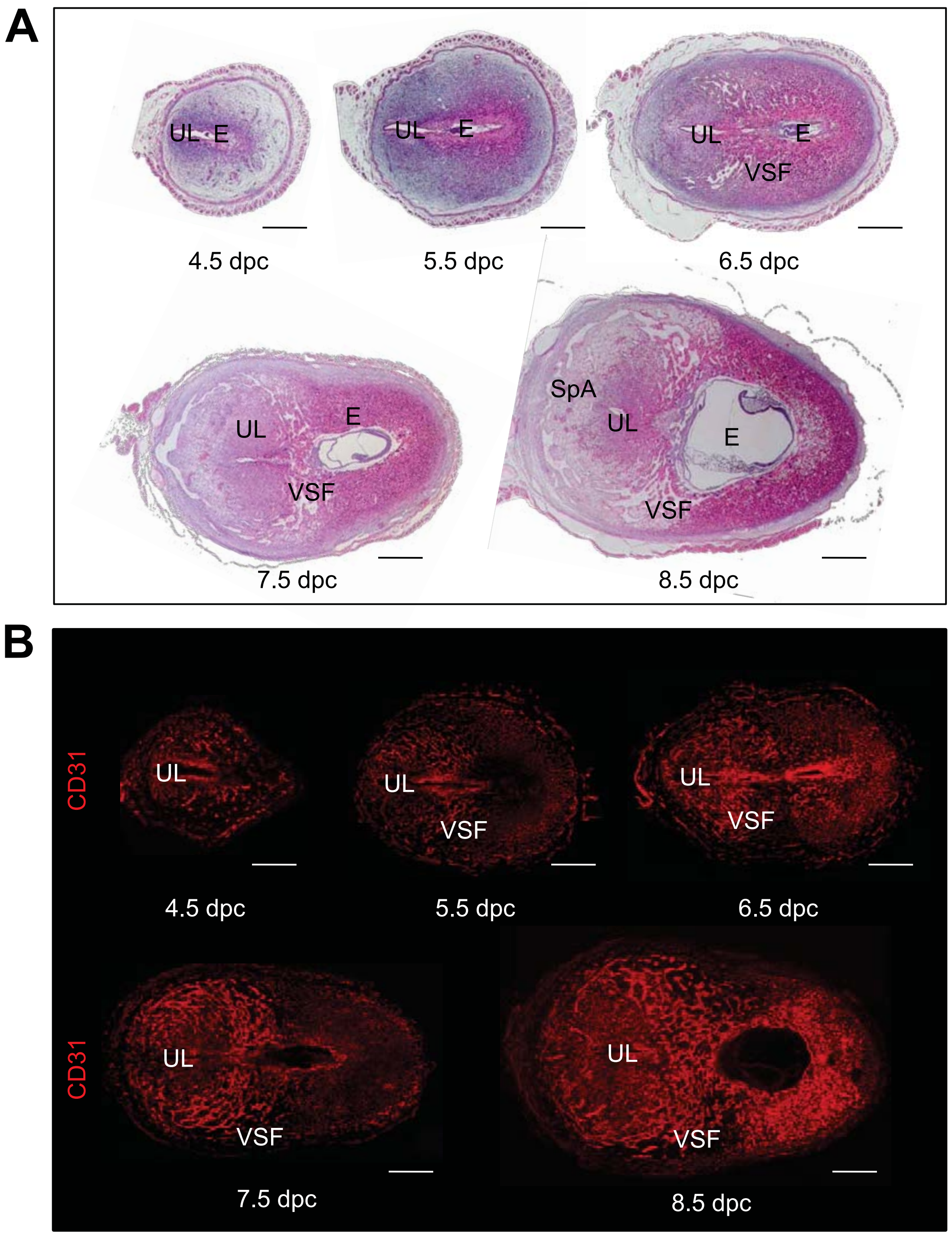

Supplementary Figure 1. Dynamic growth of uterine size and BVs during early pregnancy in mouse. (**A**) Cross sectioned uteri from 4.5 to 8.5 dpc stained with hematoxylin and eosin. UL, uterine lumen; E, embryo. (**B**) Representative images showing $CD31^+$ BVs in the uteri from 4.5 to 8.5 dpc. All scale bars, 500 μm.

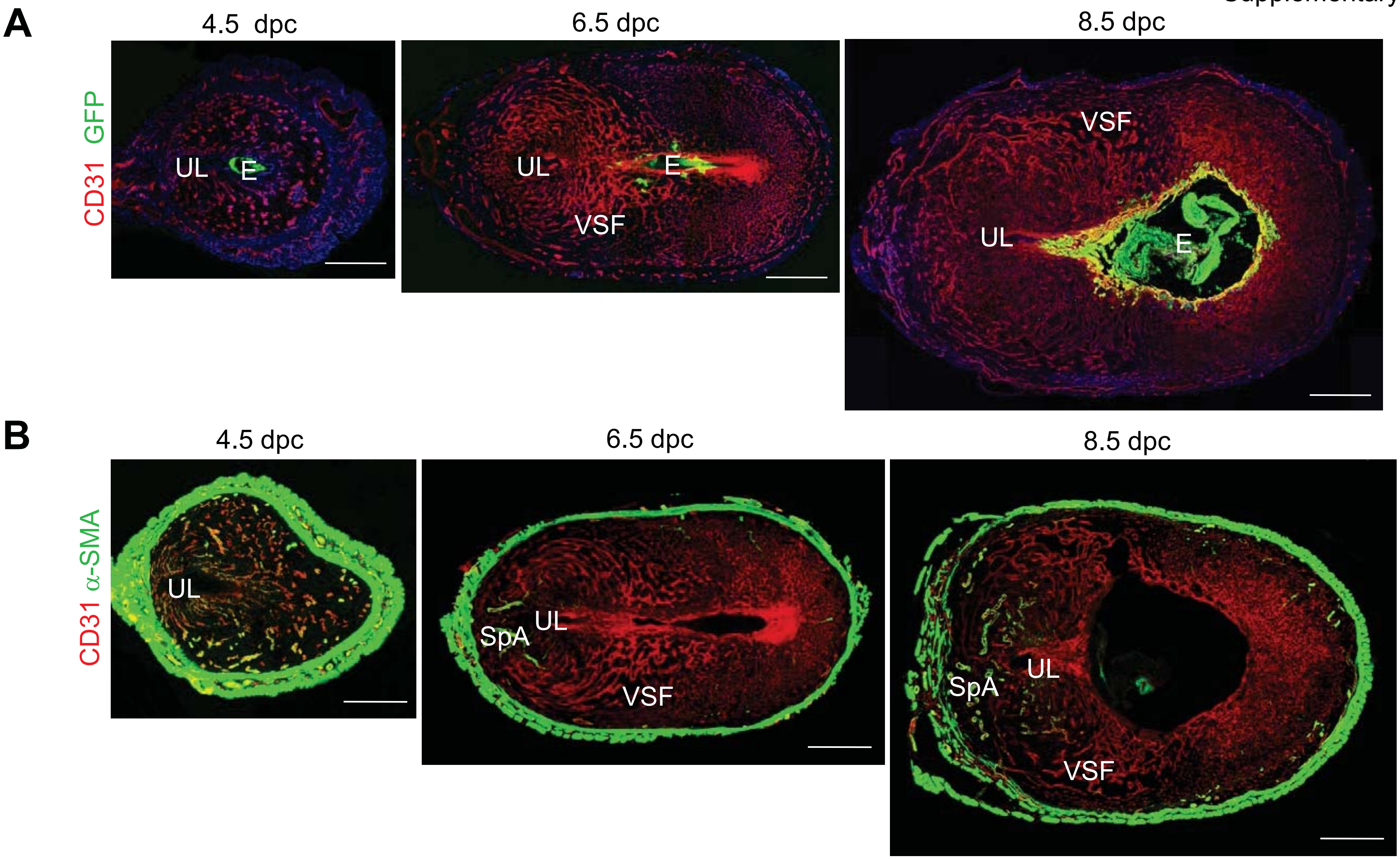

Supplementary Figure 2. Demarcation of BVs from fetus-derived placenta, and distributions of α-SMA⁺ mural cells and SpA in pregnant uteri. (A) Images showing $CD31^+$ BVs and GFP^+ embryo in the uteri at 4.5, 6.5 and 8.5 dpc. Female mice were mated with male GFP^+ mice. UL, uterine lumen; E, embryo. (B) Images showing $CD31^+$ BVs and α-SMA^+ mural cells at 4.5, 6.5 and 8.5 dpc. All scale bars, 500 μm. SpA, spiral artery.

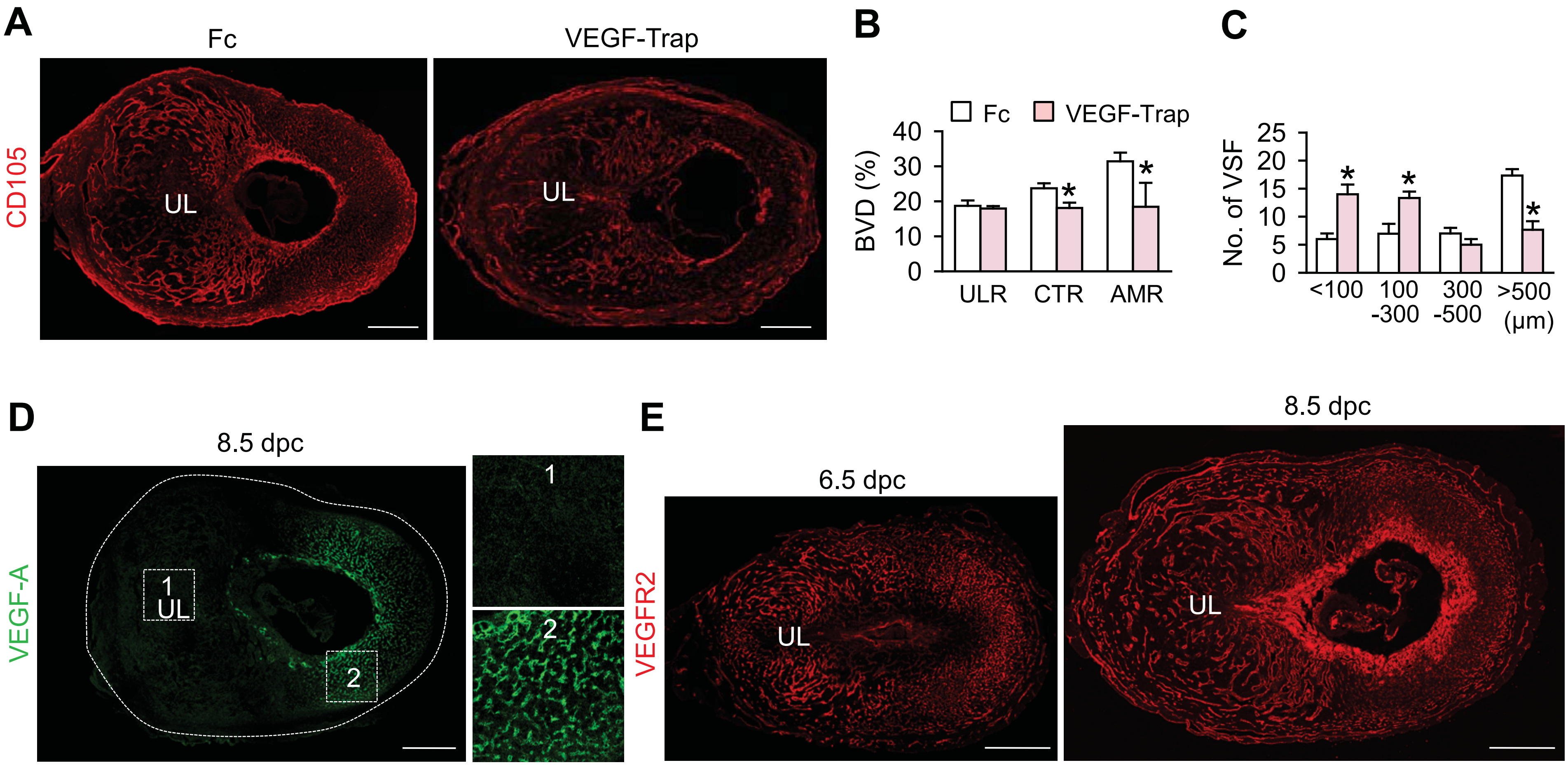

Supplementary Figure 3. Role of VEGF-A in decidual angiogenesis and EEVSF, and immunohistochemical localizations of VEGF-A and VEGFR in the pregnant uteri. (**A**) Images showing CD105$^+$ BVs in the uteri at 8.5 dpc treated with Fc and VEGF-Trap at 6.5 dpc. (**B** and **C**) Comparisons of CD105$^+$ BVD (%) in each region, and numbers of different sized VSFs in the CTR. Each group, n = 3. *p < 0.04 versus Fc by one-way ANOVA. (**D**) Images showing VEGF-A distribution in the uterus at 8.5 dpc. (**E**) Images showing VEGFR2 distributions in the uterus at 6.5 and 8.5 dpc. Each numbered region (square-dotted line) is magnified and arrayed in right. All scale bars represent 500 μm.

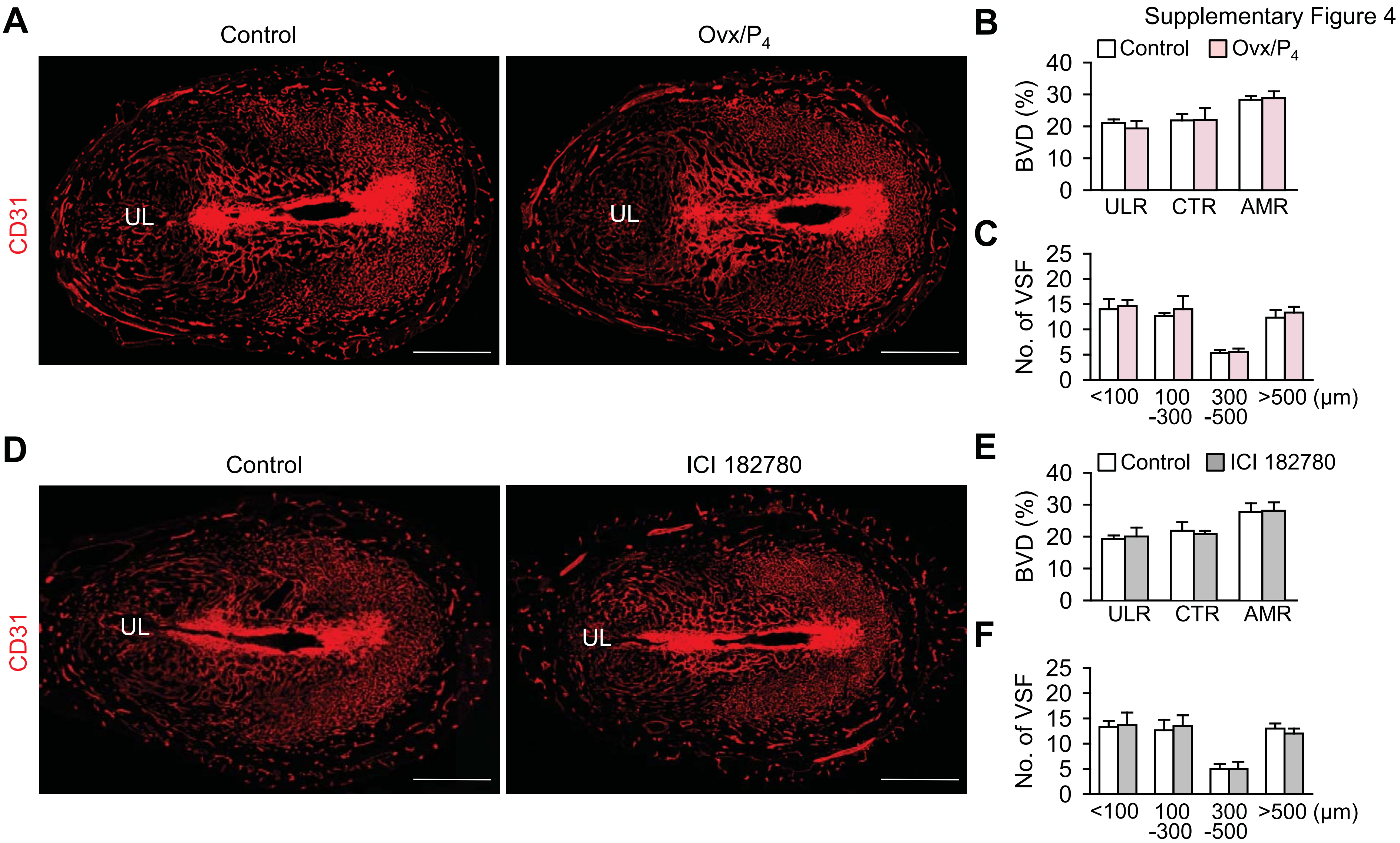

Supplementary Figure 4. Ovary-secreting E2 does not governs decidual angiogenesis and EEVSF during early post-implantation period. (**A**) Images comparing CD31$^+$ BVs in the uteri at 6.5 dpc of sham-operated Control and ovariectomized (Ovx) mice supplemented with P4 (Ovx/ P4). UL, uterine lumen. Scale bars, 500 μm. (**B** and **C**) Comparisons of CD31$^+$ BVD (%) in the ULR, CTR and AMR, and numbers of different sized VSFs in the CTR at 6.5 dpc in Control and Ovx/ P4 mice. Each group, n = 4. (**D**) Images comparing CD31$^+$ BVs in the uteri at 6.5 dpc treated with control and ICI 182780. Scale bars, 500 μm. (**E** and **F**) Comparisons of CD31$^+$ BVD (%) in the ULR, CTR and AMR, and numbers of different sized VSFs in the CTR at 6.5 dpc in control and ICI 182780. Each group, n = 4.

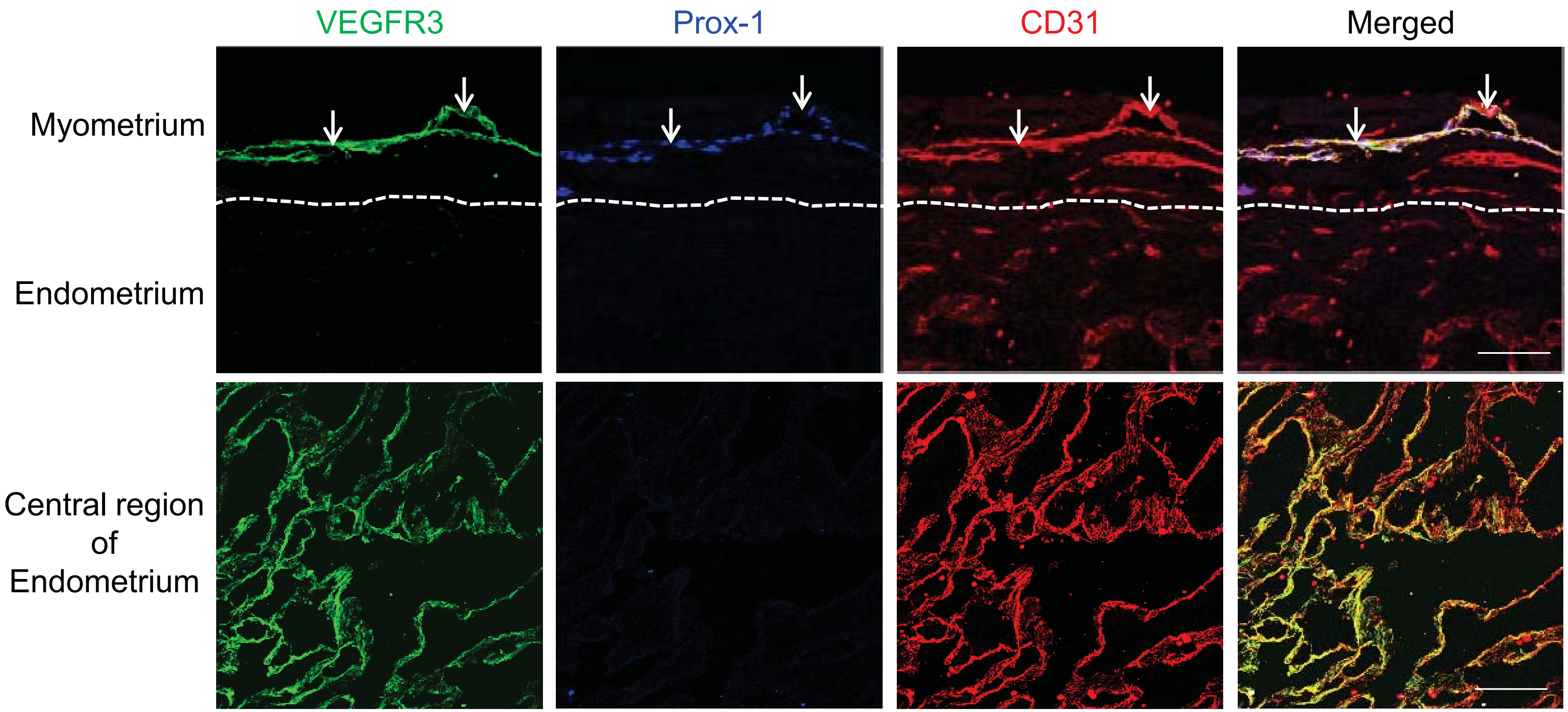

Supplementary Figure 5. Images showing VEGFR3⁺/Prox-1⁺/CD31⁺ lymphatic vessels (arrows) in the myometrium and VEGFR3⁺/Prox-1⁻/CD31⁺ VSF in the CTR of endometrium at 8.5 dpc. Scale bars, 100 μm.

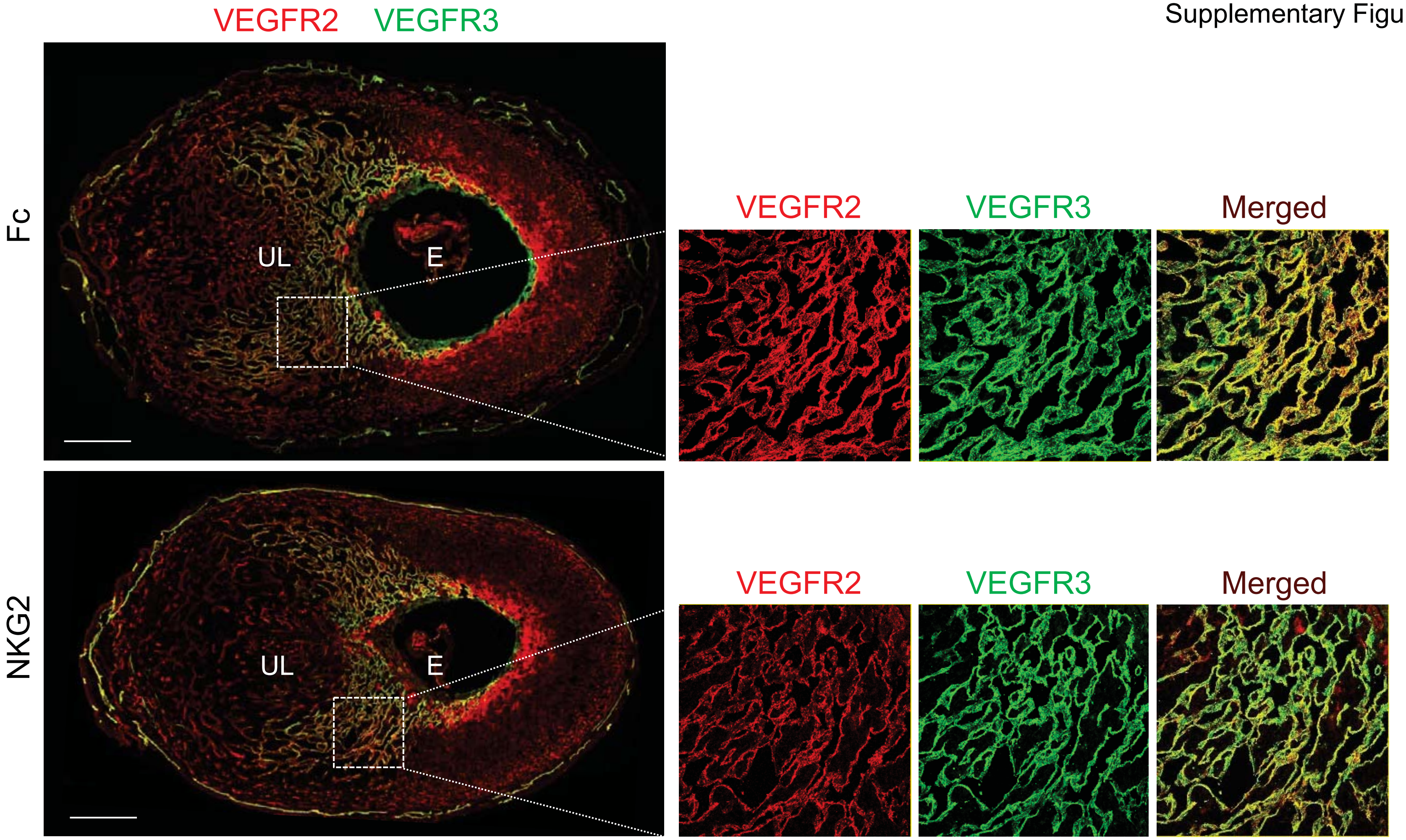

Supplementary Figure 6. Depletion of uNK cells does not reduce expressions of VEGFR2 and VEGFR3 in the vascular sinus folding of pregnant uteri. Images showing VEGFR2 and VEGFR3 expressions in the uteri at 8.5 dpc treated with Fc and anti-NKG2D antibody at 6.5 and 7.5 dpc (twice a day). Each square region (white-dotted line) is magnified in the right side. E, embryo. UL, uterine lumen. Scale bars, 500 μm. Three independent experiments show similar findings.

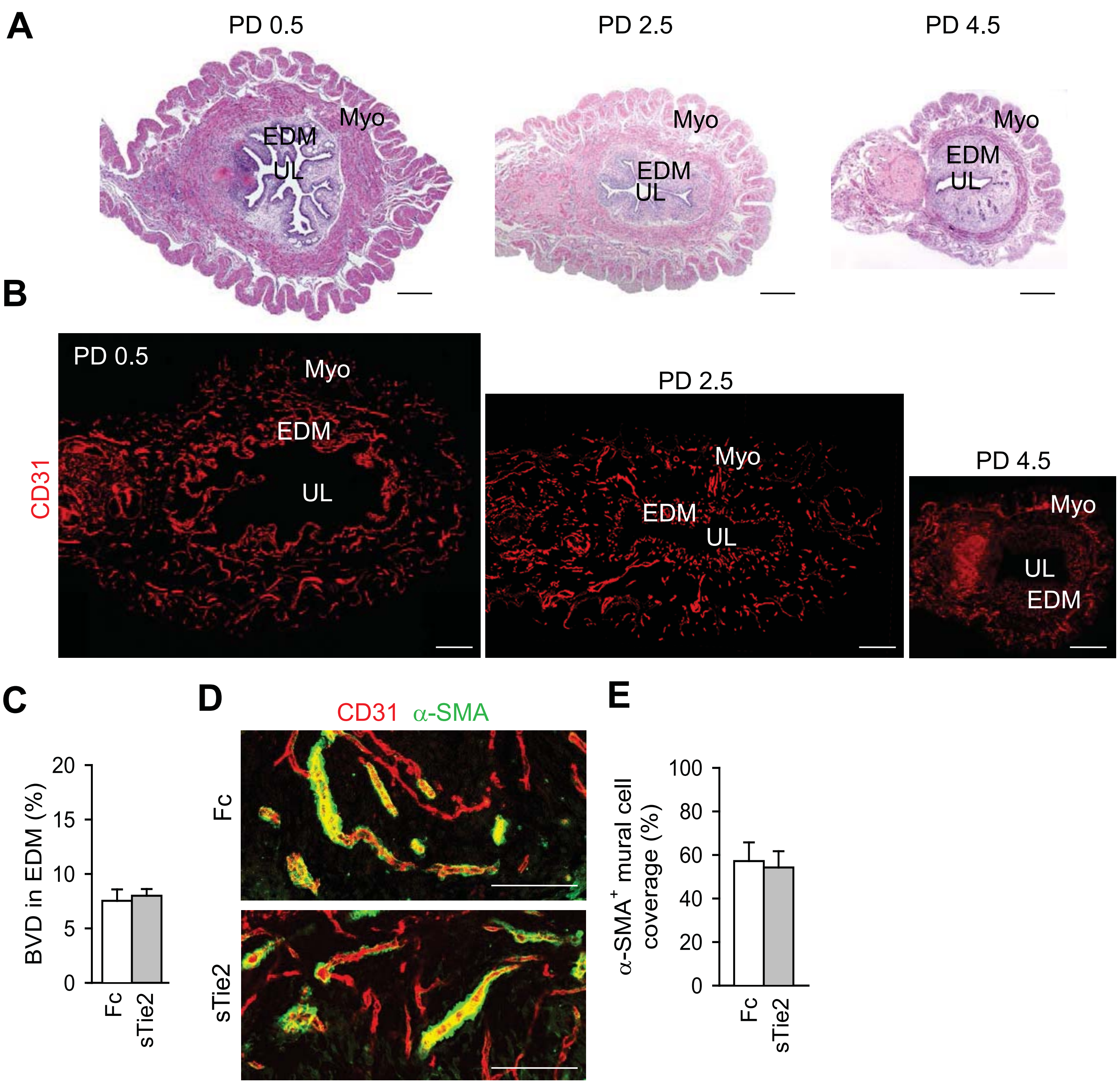

Supplementary Figure 7. Normalization of uterine size and BVs, and roles of Ang1, Ang2 and Tie2 during postpartum period. (**A** and **B**) Cross sectioned uteri at PD 0.5, 2.5 and 4.5 stained with hematoxylin and eosin and for $CD31^+$ BVs. Scale bars, 500 μm. Myo, myometrium; EDM, endometrium; UL, uterine lumen. (**C**) Comparison of $CD31^+$ BVD (%) in the endometrium at PD 2.5 treated with Fc and sTie2. Each group, n = 3. (**D**) Images showing coverage of α-SMA^+ mural cells on $CD31^+$ BVs in the endometrium at PD 2.5 treated with Fc and sTie2. Scale bars, 100 μm. (**E**) Comparison of coverage of α-SMA^+ mural cells on $CD31^+$ BVs (%) in the endometrium at PD 2.5 treated with Fc and sTie2. Each group, n = 3.

Table S1. Effects of various agents on decidual angiogenesis, EEVFS, and embryo resorption

Mouse	Agent	Dosage	Time of Administration	Time of Sacrifice	Decidual Angiogenesis EEVFS	Embryo Resorption*	n
**C57BL/6**	VEGF-Trap	25 mg/kg	4.5 dpc	6.5 dpc	Moderate impairment	42.8%	8
**C57BL/6**	VEGF-Trap	25 mg/kg	6.5 dpc	8.5 dpc	Minor impairment	<5%	3
**C57BL/6**	sVEGFR3	25 mg/kg	6.5 dpc<br>7.5 dpc	8.5 dpc	No impairment	<5%	4
**CD11c:DTR**	DT	2 ng/g	4.5 dpc	6.5 dpc	Severe impairment	100%	3
**CD11c:DTR**	DT	2 ng/g	5.5 dpc	6.5 dpc	No impairment	<5%	4
**CD11c:DTR**	DT	2 ng/g	6.5 dpc	8.5 dpc	No impairment	<5%	3
**C57BL/6**	NKG2D	7.5 mg/kg	6.5 dpc<br>7.5 dpc	8.5 dpc	Minor impairment	<5%	4
**C57BL/6**	P4	25 mg/kg	4.5 dpc<br>5.5 dpc	6.5 dpc	Enhanced	<5%	4
**C57BL/6**	RU486	8 mg/kg	5.5 dpc	6.5 dpc	Moderate impairment	28.5%	12
**C57BL/6**	ICI 182780	1 mg/kg	5.5 dpc	6.5 dpc	No impairment	<5%	4

*Embryo resorption is defined as undersized and abnormal shaped conceptus.
